# Photothermal nanohybrid hydrogels for biomedical applications

**DOI:** 10.3389/fbioe.2022.1066617

**Published:** 2022-11-03

**Authors:** Fan Ding, Linlin Zhang, Xu Chen, Weiling Yin, Li Ni, Miao Wang

**Affiliations:** ^1^ Institute for Advanced Materials, School of Materials Science and Engineering, Jiangsu University, Zhenjiang, Jiangsu, China; ^2^ Department of Orthopaedic Surgery, Orthopedic Institute, The First Affiliated Hospital, Suzhou Medical College, Soochow University, Suzhou, Jiangsu, China

**Keywords:** biomedical hydrogel, photothermal therapy, photothermal antibacterial, photothermal cancer suppressor, photo-controlled drug release

## Abstract

In the past decades, diseases such as wound infection, cancer, bone defect and osteoarthritis have constantly threatened the public health. However, the traditional treatment has many insufficiencies, such as high cost, easy recurrence and high biological toxicity. Hydrogel is a material with three-dimensional network structure, which has a series of advantages, such as injectability, self-heal ability, easy loading and controllability of drug release, and excellent biocompatibility. Therefore, it is extensively used in drug delivery, antibacterial, anti-cancer and other fields. However, the traditional hydrogels have the single performance, and therapeutic efficacy is often rely on the drugs loaded on them to cure diseases, which cannot achieve sustainable therapeutic effect. In order to solve this problem, photothermal nano hydrogel with photothermal agent (PTA) has become an ideal material due to its excellent physical and chemical properties. Photothermal nano hydrogels used in photothermal therapy (PTT) can exploit the photothermal effect of photothermal agent to increase local temperature and control the sol-gel phase transition behavior of hydrogels, so they are widely used in drug release, photothermal sterilization, photothermal inhibition of cancer cells and enhancement of bone repair. To sum up, this paper introduces the preparation of hydrogels with photothermal nanomaterials, and discusses their applications in the fields of drug release, photothermal sterilization, photothermal cancer cell inhibition and enhanced bone repair.

## Introduction

Hydrogel is a kind of soft material with a three-dimensional network structure, which is made up of networks with hydrophilic polymers, and is crosslinked by physical or chemical bonds between strong water absorption. By simulating the composition, physical and chemical properties of the natural extracellular matrix (ECM), the hydrogel performs good biodegradability and biocompatibility. (Cao et al., 2021; Pei et al., 2021). And the hydrogels exhibit stimulus response and self-healing properties under the stimulation of external environment can meet the needs of hydrogels in medical materials, which has attracted extensive attention of researchers (Ou & Tian, 2021; Xie et al., 2021; Zhang & Lucia, 2021). In recent years, hydrogels have been widely used in biomedical fields, such as drug delivery, antibacterial therapy, biosensors and cancer cell inhibition (Xu et al., 2022a; Xu et al., 2022b; Zhang et al., 2022).

Photothermal nano hydrogel is a kind of hydrogel with photothermal nano materials added during the preparation of hydrogel. Photothermal therapy (PTT) generated by photothermal nano hydrogel is a typical photon triggered therapy method. It can use the photothermal effect of photothermal agent (PTA) to extract energy from visible light/near-infrared light, convert it into heat, increase the temperature of the surrounding environment, and achieve the effect of ablating of tumor cells and killing bacteria. (Chen et al., 2021; Guedes et al., 2021). It is highly necessary to choose the appropriate photothermal agent. The ideal photothermal agents with appropriate NIR band gap and high response to near-infrared light irradiation can effectively convert light energy into heat energy under near-infrared light irradiation, and improve the therapeutic effect (Lu Y. et al., 2021). Photothermal treatment has many advantages. It can reduce the pain of patients during treatment. Secondly, it has short processing time and obvious therapeutic effect. More importantly, the materials used for photothermal treatment are of low toxicity or even non-toxic, causing less harm to the human body. So far, many forms of photothermal therapy have been studied and applied to the field of anti-cancer and antibacterial (Yu et al., 2019; Zhao et al., 2022).

Photothermal agent (PTA) is an crucial factor of photothermal therapy and the selection of appropriate photothermal agent is very important to the success of photothermal therapy (Guedes et al., 2021; Zhang et al., 2021). PTA should have high photothermal conversion efficiency, easy to prepare and good biocompatibility. However, many photothermal agents with high thermal efficiency have certain toxicity, which is not suitable for medical application. Hydrogels made by combining materials with photothermal agents with high biocompatibility can not only retain the high photothermal conversion effect, but also reduce biological toxicity and make them more biocompatible. In this paper, several typical photothermal nanohydrogels are reviewed and their applications in biomedical fields are also discussed.

## Photothermal nanomaterials for hydrogel fabrication

The preparation of photothermal nanohydrogels mainly relies on the photothermal nanomaterials. The most widely used ones mainly include Metal nanomaterials, Carbon based nanomaterials, Metal sulfide/oxide nanomaterials, Black phosphorus nanomaterials, MXenes nanomaterials, Polymer nanomaterials, Organic dye nanomaterials, etc. In the following, the development of different photothermal materials for the preparation of photothermal nanohydrogels will be discussed in detail.

### Metal nanomaterials

The properties of metal nanomaterials are very excellent. It has a strong surface plasmon resonance (LSPR) effect. When the incident photon frequency matches the overall vibration frequency of the metal nano material, the nano material will have a strong absorption effect on the photon energy, and a strong resonance absorption peak will appear in the spectrum. (Ai et al., 2021). Moreover, metal nanomaterials have excellent thermal properties, high absorption cross section and high field conversion efficiency in the near infrared region. Metal nanomaterials combine with peptides, antibodies, biocompatible polymers, chemical drugs and immune factors, and have great potential in the field of biomedicine (Park et al., 2018). The metal nanomaterials most explored and studied in PTT are gold nanomaterials, silver nanomaterials and copper nanomaterials, All of them have the advantages of strong absorption, excellent adjustable physical properties, optical properties and biocompatibility (Xu H. et al., 2020; Lu X. et al., 2021).

**FIGURE 1:**
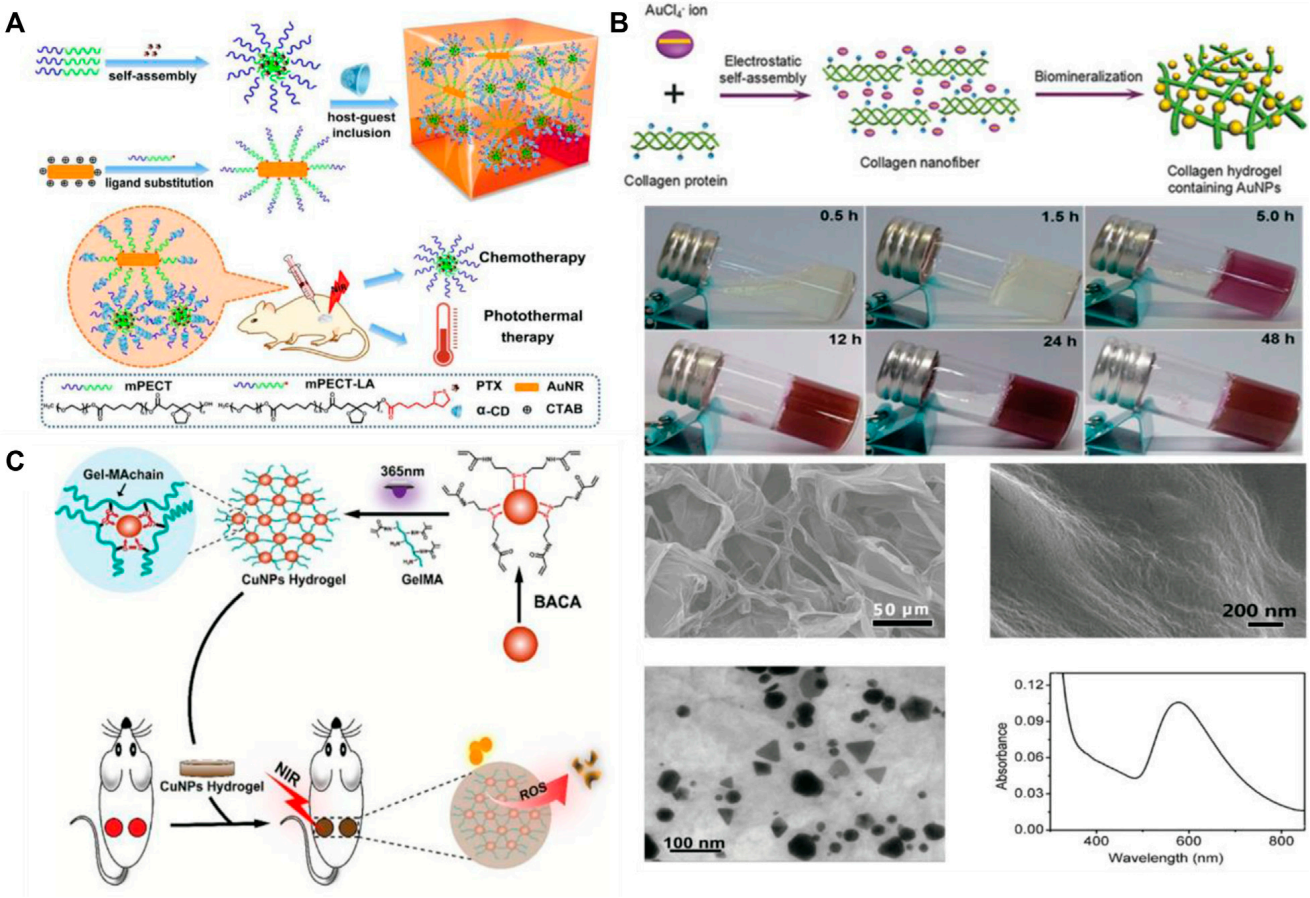
**(A)** Preparation of gold nano hydrogel and its application in chemotherapy-assisted photothermal therapy. Reproduced from ref.49 with permission from 2019 Royal Society of Chemistry. **(B)** Synthesis and characterization of injectable and self-healing collagen hydrogels containing gold nanoparticles. Reproduced from ref. 87 with permission from 2016 WILEY-VCH. **(C)** Preparation of copper nano hydrogel and schematic diagram of antibacterial principle of photothermal therapy. Reproduced from ref. 75 with permission from 2019 Royal Society of Chemistry.

At present, various configurations of nanostructures based on gold have been developed. Among them, gold nanorods (GNR) have attracted much attention because of their simple biological coupling, strong and adjustable plasma absorption. In particular, there are two plasmon resonance surfaces on the surface of GNR, the transverse band represented in the visible region (650 nm–950 nm) and the longitudinal band represented in the near infrared region (1000 nm–1350 nm), so the radiation can penetrate tissues to the maximum extent, making it an ideal material for biomedical applications (Zhang Y. et al., 2020; Gupta & Malviya, 2021). GNR have biofilm activity and are an attractive therapeutic method for photothermal therapy. The hydrogel added with gold nanoparticles (GNP) shows certain advantages in biomedical applications. On the one hand, the hydrogel has good biocompatibility and degradability. On the other hand, the GNP are used as light absorbers, making the hydrogel well used in photothermal therapy. Bermudez-Jimenez et al. prepared gold nanorod hydrogels by embedding GNR into a non-toxic, biocompatible and biodegradable chitosan hydrogel (Bermudez-Jimenez et al., 2020). Combined with PTT treatment, it can effectively control the pathogenic bacteria in the mouth. In another study, Liu et al. modified gold nanorods by a two-block copolymer, an injectable nanocomposite hydrogel was prepared by the interaction of α-cyclodextrin (Figure 1A)(Liu M. et al., 2019). The hydrogel can not only improve the biocompatibility of AuNR, but also realize local photothermal treatment. Moorcroft et al. co loaded IRIKIRIKCONH_2_ (IK8) and GNR into polyethylene glycol (PEG) hydrogels, and achieved the bactericidal effect on *Staphylococcus aureus* by photothermal triggering the release of IK8 (Moorcroft et al., 2020). At the same time, relevant experiments further confirmed that the hydrogels loaded with GNR had certain photothermal damage to the biofilms. At present, photothermal ablation (PTA) based on nanotechnology, as a highly effective treatment method for solid tumors, has been widely explored. Gold nanoparticles, as strong light absorbers, can absorb NIR and achieve local fever through photothermal conversion effect, which can reduce the damage to tissues around the wound to the maximum extent while treating the wound (Zhang R. et al., 2020). Xing et al. proposed a method based on biomineralization trigger for the first time to prepare collagen hydrogels with adjustable mechanical properties (Figure 1B) (Xing et al., 2016). Through electrostatic bonding between collagen chains (positively charged) and inorganic anion clusters, GNP were formed, which were controlled as cross-linking agents for mechanical properties, to prepare hydrogels with advantages of *in vivo* injection. When the stress relaxation of the hydrogel is caused by the non-covalent interaction between GNP and collagen chains, the hydrogel can recover rapidly under the condition of applied stress. This study expands the application of GNP hydrogel.

Silver nanoparticles (Ag NP_s_) have similar photothermal properties to GNP. Because of their photothermal effects, toxicity of silver, and wide applicability in the pharmaceutical field, they have always attracted the attention of researchers. (Awasthi et al., 2020; Ren et al., 2021). Silver nanoparticles have become a good photothermal agent because of their high photo thermal conversion efficiency, easy synthesis and multifunctional adjustability of their surface properties (de Oliveira Lima et al., 2022). Recently, Amatya et al. prepared hydrogel films with good photothermal activity through bovine serum protein (BSA) and Ag NPs and applied them *in vivo* (Amatya et al., 2021). Under 0.6 W low laser power, the temperature can be reached 45 °C which is ample for tumor ablation.

Due to local surface plasmon resonance, copper nanoparticles show strong light absorption in visible and near infrared, similar to that of silver and gold nanoparticles. Nano copper has been widely used in wound healing due to its high redox potential, low production cost and broad-spectrum antibacterial activity. Chen et al. successfully embedded nanoparticles into guar gum hydrogel to form copper nanoparticle hydrogel (Chen et al., 2017). The copper nanoparticles embedded in the hydrogel have a good photo thermal conversion rate. After 10 min of laser irradiation, the temperature of the hydrogel can rise to 67°C. This rapidly rise in temperature contributes to the high antibacterial performance of copper on irradiated nanoparticle hydrogels. Tao et al. reported a MA modified copper nanoparticle hydrogel (Figure 1C) (Tao et al., 2019). In combination with 808 nm NIR radiation, copper NPs embedded in the hydrogel can produce reactive oxygen species (ROS), and effectively convert NIR laser energy into local heat. It can eradicate *Escherichia coli* and *Staphylococcus aureus* bacteria *in vitro* antibacterial experiments. Most importantly, the hydrogel can also promote wound healing and realize multi-functional application of hydrogel.

### Carbon-based nanomaterials

**FIGURE 2:**
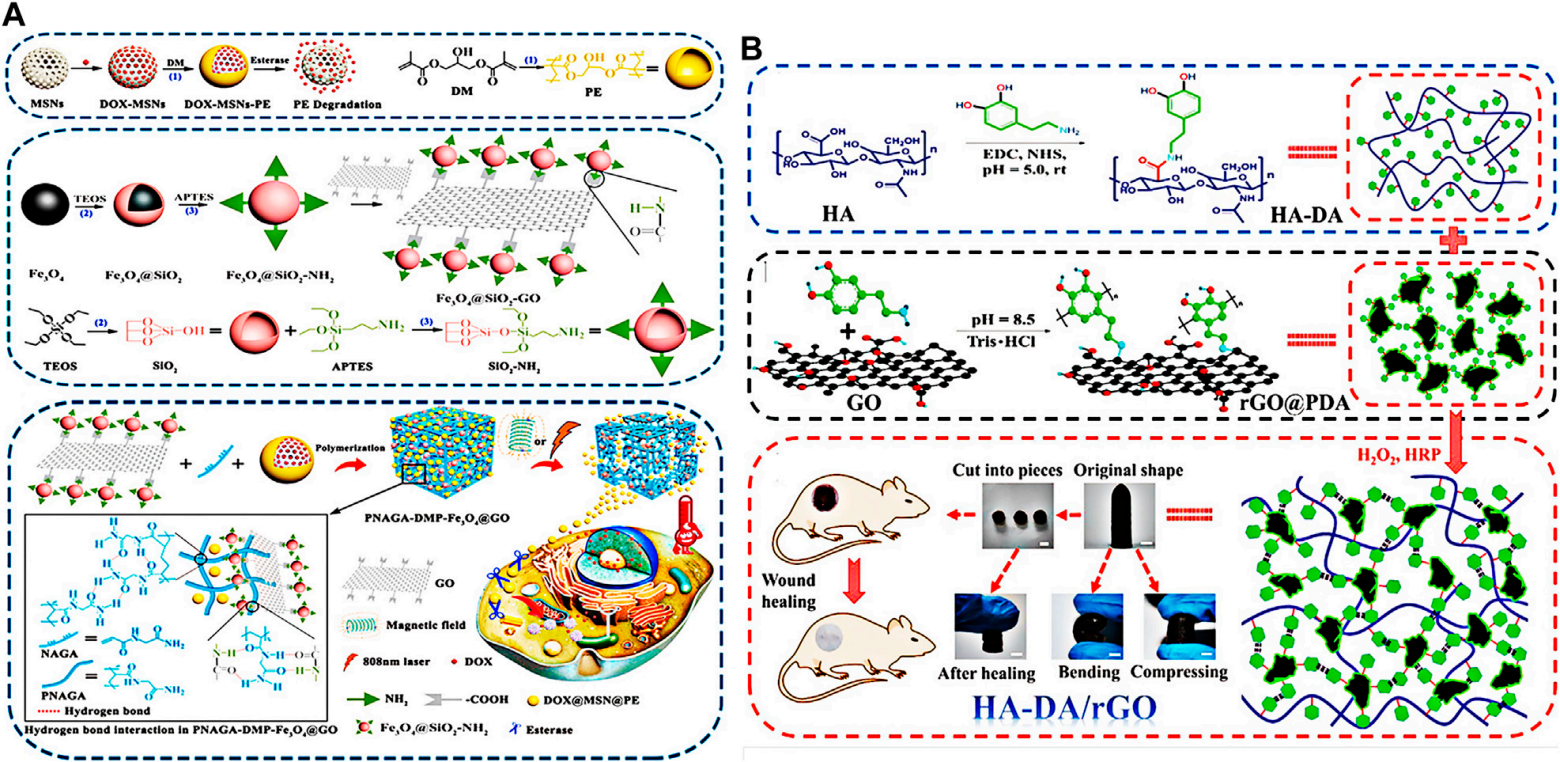
**(A)** Schematic diagram of the preparation of nanocomposite hydrogel and its mechanism in tumor therapy. Reproduced from ref. 101 with permission from 2020 Elsevier ltd. **(B)** Diagrammatic sketch of HA-DA/rGO hydrogel preparation. Reproduced from ref. 42 with permission from 2019 WILEY-VCH.

Graphene is a new material with a single-layer two-dimensional honeycomb lattice structure, which is closely packed with sp^2^ hybrid connected carbon atoms (Deng X. et al., 2020). The sp^2^ hybrid carbon atoms of Graphene oxide in the hexagonal lattice structure allows to absorb light of different wavelengths, and its photothermal capacity can be enhanced with the increase of photoabsorption, which make it a good photothermal heating material (Falke et al., 2020; Huang, 2022). Lee et al. used graphene oxide and modified it with polyethylene glycol to develop a wavelength independent hydrogel system, to improve the dispersion of graphene oxide in aqueous solution (Lee et al., 2020). Under the irradiation of 532 nm, 785 nm and 980 nm lasers, the temperature of graphene oxide polyethylene glycol solution can reach 43°C, and free radical polymerization can be triggered at this temperature. Yuan et al. prepared a magnet and light double response hydrogel by introduced Fe_3_O_4_-GO nanocomposite as a magneto photothermal agent. Fe_3_O_4_-GO nanocomposite can convert the external magnetic field and near-infrared (NIR) light into heat, which can effectively improve the local temperature in the hydrogel (Figure 2A) (Yuan et al., 2020). Li et al. modified graphene oxide with polyethyleneimine, an amine terminal polymer branch, and prepared a hydrogel (Li et al., 2019). The hydrogel is not only structurally stable, but also can provide continuous drug delivery and near-infrared photothermal effect. Also many researchers studied the reduction of graphene oxide to improve the photothermal properties of graphene oxide. Liang and his colleagues prepared a series of hyaluronic acid grafted dopamine and reduced graphene oxide (rGO) hydrogels using the H_2_O_2_/HPR (horseradish peroxidase) system (Figure 2B) (Liang et al., 2019). The hydrogels have good self-healing properties, antioxidant activity and tissue adhesion. And most importantly, the enhanced antibacterial properties of the hydrogels through near-infrared (NIR) radiation, make them a good wound dressing. Liu and his colleagues prepared the hydrogel by functionalizing and reducing graphene oxide with pH responsive carboxymethyl chitosan (Liu W. et al., 2019). The hydrogel not only has excellent degradability and biocompatibility, but also has better photothermal conversion efficiency than many other photosensitizers, reaching 86.7%.

### Metal sulfide/oxide nanomaterials

Metal sulfides/oxides which cost less than precious metals are also use in PTT. Copper sulfide nanostructures have excellent photothermal properties. Unlike the infrared absorption of gold nanostructures, the infrared absorption of copper sulfide nanoparticles comes from the energy band transition (Sun et al., 2019; Xie et al., 2022). Fu and his colleagues reported an injectable and thermosensitive hydrogel encapsulating copper sulfide nanoparticles (Fu et al., 2018). Nanodots are uniformly distributed in the hydrogel matrix, and their particle size remains unchanged. The hydrogel not only shows the ability of forming *in-situ* gel with thermal response, and the chemical toxicity of copper sulfide was reduced by “composing” nano dots in the matrix. In another study, Lin et al. incorporated copper sulfide nanoparticles (CuS NPs) into hyaluronic acid (HA) to construct hydrogels (Lin et al., 2021). By combining the photothermal characteristics of CuS NPs, the sterilization of low temperature photothermal therapy is realized, also the improvement of the antibacterial efficiency and minimization of the damage to normal tissues. The team combined the photothermal effect and antibacterial effect provides a new idea for the new type of wound bandage.

Silver sulfide quantum dots are semiconductor materials with strong light stability and high biocompatibility. Because of their unique properties such as broadband absorption, convenient preparation, good chemical stability and low toxicity, they have attracted extensive attention. Recently, Hou et al. encapsulated the near-infrared silver sulfide quantum dots as photosensitizers in the hydrophobic cavity by self-assembly of polypeptide hydrogels, and then integrated the drugs DOX and Bestin into the hydrogels, thus prepared a multifunctional gene engineering polypeptide hydrogel encapsulating silver sulfide quantum dots (Hou et al., 2020). Due to the photothermal properties of silver sulfide quantum dots, the release of DOX from hydrogels is promoted, thus the overall therapeutic effect is improved.

**FIGURE 3:**
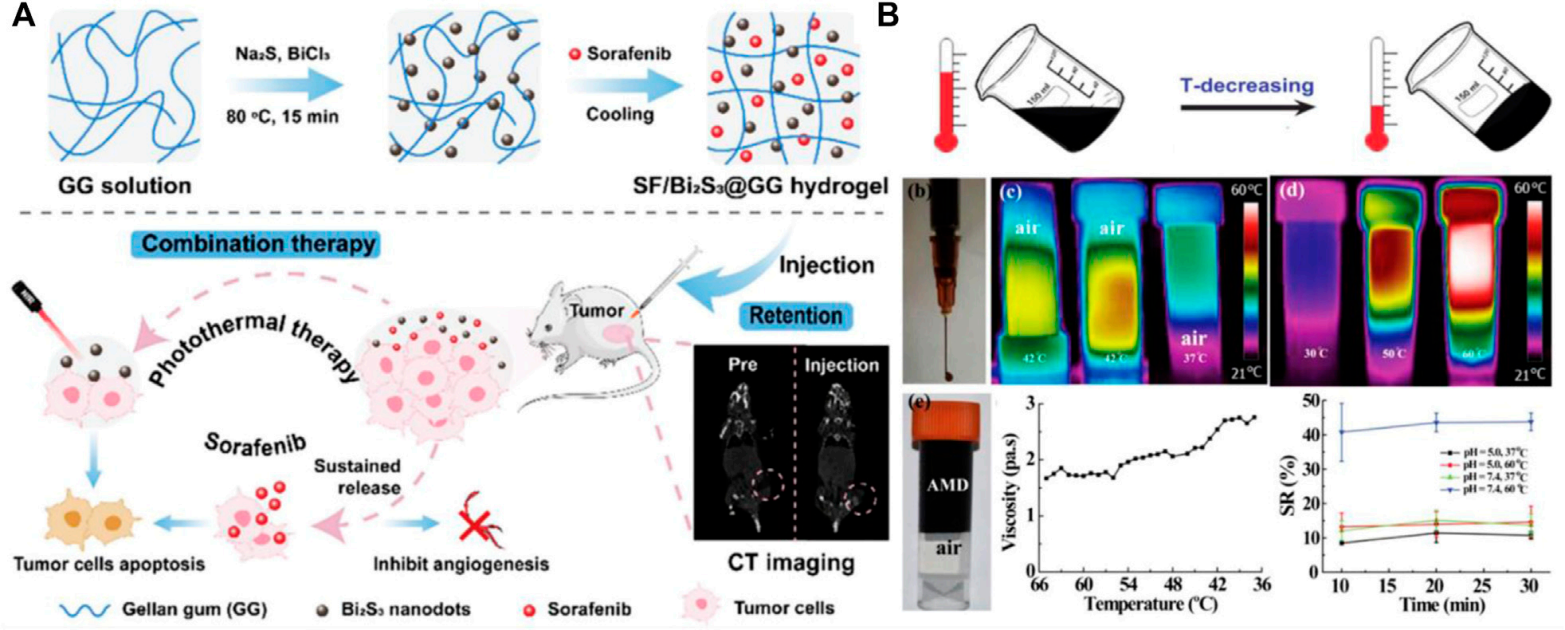
**(A)** Preparation of SF/ Bi2S3@GG photothermal nanohydrogel and its application in tumor therapy. Reproduced from ref. 83 with permission from 2021 Royal Society of Chemistry. **(B)** The formation principle of AMD hydrogel and its photothermal performance test. Reproduced from ref. 81 with permission from 2017 Published by Elsevier Ltd.

Bismuth sulfide (Bi_2_S_3_) is a promising PTT agent with a narrow direct band gap (E ≈ 1.3 eV). Bi_2_S_3_ nanostructures have been used as CT contrast agents, its cost is much lower than other metal elements (such as gold, platinum and tantalum). Bi_2_S_3_ nanoparticles also have biocompatibility and metabolism, and have low toxicity. Different types of Bi_2_S_3_ nanoparticles, such as Bi_2_S_3_ nanorods, nano porous bladder and nanodots are more commonly used. Smaller Bi_2_S_3_ nanoparticles are thought to have better light absorption and can be excreted from the bladder. Wu et al. embedded the ultra-small (less than 3 nm) Bi_2_S_3_ nano point into the hydrogel to improve the stability of the Bi_2_S_3_ nano point and endow the injectability of hydrogel (Figure 3A) (Wu et al., 2021). The hydrogel can maintain the same photothermal performance after being stored for 3 months. In another study, Wu et al. designed and synthesized MoS_2_/Bi_2_S_3_-PEG (MBP) nano sheets (Figure 3B). And they dispersed them together with DOX into agar solution to build a hydrogel system with photothermal conversion performance, and achieve tumor PTT and chemotherapy under the guidance of computer tomography (CT)/photoacoustic (PA) dual model imaging (Wu et al., 2018).

MoS_2_ is another representative sulfide. Jin et al. designed to load positively charged DOX and negatively charged PC10A onto the surface of molybdenum disulfide nano sheets to prepare mixed PC10A/DOX/MoS_2_ nano particles and dispersed them in hydrogels (Jin et al., 2020). Molybdenum disulfide nano sheets were used as both photothermic agent and photodynamic agent in hydrogels. The production of hot oxygen and reactive oxygen can cause immune response and promote photothermal therapy on tumors. Zhou et al. reported a simple method to prepare sodium alginate (ALG) - Fe^3+^(MAF) hydrogel containing molybdenum disulfide and glucose oxidase (GOx) (Zhou L. et al., 2020). The hydrogel has high photothermal conversion capacity of molybdenum disulfide, and an enzymatic reaction could occur in the hydrogel, which provides an effective way for the use of enzymes in cancer treatment.

### Black phosphorus nanomaterials

**FIGURE 4:**
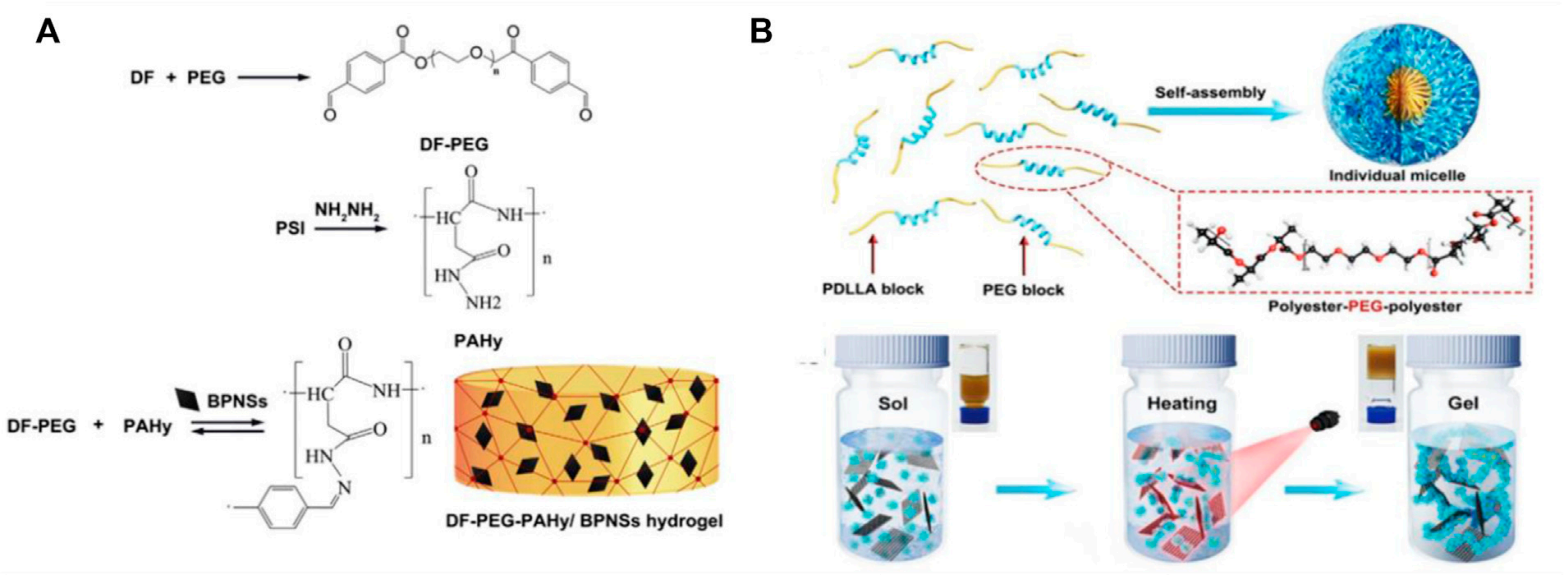
**(A)** Schematic diagram of preparation of black photothermal nanohybrid hydrogels. Reproduced from ref. 82 with permission from 2019 Published by Elsevier Inc. **(B)** Preparation of black phosphorus nano hydrogel and its schematic diagram of gel sol transition under infrared light irradiation. Reproduced from ref. 70 with permission from 2018 The Authors. Published by WILEY-VCH.

Black phosphorus nanomaterials (BP) nano sheet is a kind of two-dimensional nano material with unique properties such as adjustable band gap, high NIR absorption and high photo thermal conversion efficiency (Eswaraiah et al., 2016; Ren et al., 2017). BP nano sheet has the characteristics of highly efficient single oxygen generation, and has extensive NIR absorption and photothermal conversion characteristic under whole visible light region, and is extensively used in photothermal therapy. As an inorganic nano agent, BP nano tablets are attractive due to their biocompatibility. Because phosphorus is an important element in human bones, accounting for about 1% of human body weight. Qin et al. used the biocompatible copolymer F127 as the matrix to construct the thermosensitive hydrogel together with the photothermal therapeutic agent BP nano sheet (Qin et al., 2019). The hydrogel has the characteristics of near infrared photothermal conversion, photothermal stability and biodegradability. Wu et al. prepared a pH sensitive DF-PEG-PAHy/BPNSs hydrogel by adding black phosphorus nanoparticles (BPNSs) into the hydrogel formed by diphenylaldehyde functionalized polymer and polyaspartic hydrazine polymer (Figure 4A) (Wu et al., 2019). This study shows that the hydrogel has good gel characteristics, pH sensitivity and near-infrared response. Due to the photothermal effect of BP NPs, NIR accelerates the release of drugs in the hydrogel. In addition, BP nano tablets are naturally degraded in the physiological environment, in the form of harmless PO_4_^3-^ as the final degradation product. Shao and his colleagues combined BP nano tablets with thermosensitive hydrogels to prepare hydrogels for photothermal therapy after cancer surgery (Figure 4B) (Shao et al., 2018). The research shows that the hydrogel has excellent NIR PTT performance, good biodegradability and biocompatibility. It can promote the rapid transformation of sol gel under NIR irradiation safely. A gel film can quickly form by spraying the hydrogel under NIR irradiation on the wound, which performed a high PTT effect and can eliminate the residual tumor tissue.

### MXenes nanomaterials

**FIGURE 5:**
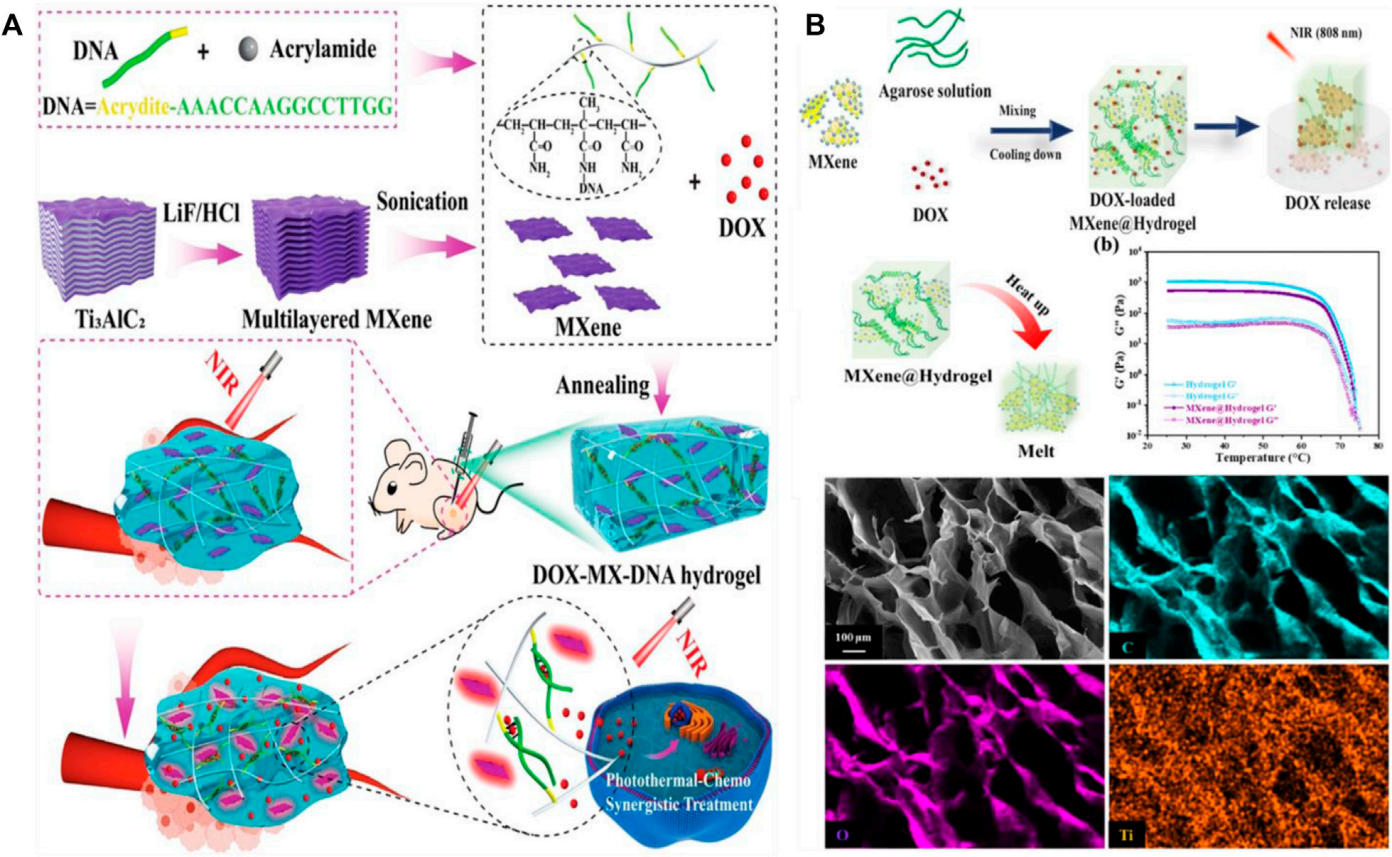
**(A)** Schematic diagram of the principle of MXene-DNA hydrogel loaded drugs in cancer treatment. Reproduced from ref. 29 with permission from 2022 Wiley-VCH GmbH. **(B)** Schematic diagram of synthesis and melting after heating of MXene based photothermal hydrogel. Reproduced from ref. 16 with permission from 2021 Published by Elsevier B.V.

After the discovery of titanium carbide (Ti_3_C_2_T_x_) by Naguib et al., in 2011, transition metal carbides, nitrides, and carbon nitrides (often referred to as MXene) have attracted widely attention because of their unique planar structures, excellent physicochemical properties, and chemical diversity (Naguib et al., 2011). The general formula for these materials is M_n+1_X_n_T_x_ (n = 1, 2, or 3), where M is an early transition metal, X is carbon and/or nitrogen, and T is the surface end inherited from the synthesis process, like -OH, -O, and -F (Xu D. et al., 2020). As a photothermal agent, MXene nanosheets exhibit strong light absorption in the near infrared range, high specific surface area and negative charge, which make abundant anchoring position of the therapeutic agent, so MXene nanosheets are widely used in photothermal therapy (Lin et al., 2017; Lin et al., 2018). He and his colleagues used MXene as photothermal agent and doxorubicin as loading chemotherapy agent, and combined it with DNA hydrogel to establish a photothermal-chemical synergistic therapy system for highly effective local cancer treatment (Figure 5A) (He et al., 2022). Under local near-infrared light irradiation, the MXene nanosheet converts light energy into heat energy and triggers the reversible transformation of hydrogel from gel to solution, releasing DOX therapeutic agent. The experimental results showed that the hydrogel had excellent biocompatibility and showed effective local cancer treatment. Dong and colleagues prepared a drug-loaded MXene/ agarose hydrogel (Dong et al., 2021). They first prepared a two-dimensional MXene nanosheet with high photothermal conversion efficiency and photothermal stability, then introduced the MXene nanosheet into the low melting point agarose gel skeleton. The temperature of the loaded hydrogel can rapidly rise to 60°C under near-infrared light and hydrolyze to release the encapsulated drug (Figure 5B). The kinetics of drug release can be regulated by agarose concentration, MXene concentration, irradiation intensity and irradiation time. This research provides a new way to develop smart hydrogel-based drug delivery systems for local cancer treatment. Li et al. prepared an anisotropic MXene@PVA hydrogel by directed cryoassisted salting-out. (Li Y. et al., 2022). Because of the good photothermal properties of MXene, the hydrogel can be used in local hyperthermia treatment of the infected site under near-infrared laser (808 nm) irradiation. In addition, the hydrogel has excellent mechanical properties, with stress up to 0.5 MPa and strain up to 800%. Bacterial experiments showed that the hydrogel had broad spectrum antibacterial activity against both Gram-positive and Gram-negative bacteria. Li and colleagues designed a hydrogel film with MXene nanosheets embedded with heat-responsive gelatin (Li Y. et al., 2022). They used an epithelial cell adhesion molecule antibody to modify the hydrogel membrane so that it could specifically recognize and isolate CTCS from whole blood. The captured CTCS can be released without damage through temperature responsive release and photothermal site release.

### Polymeric nanomaterials

**FIGURE 6:**
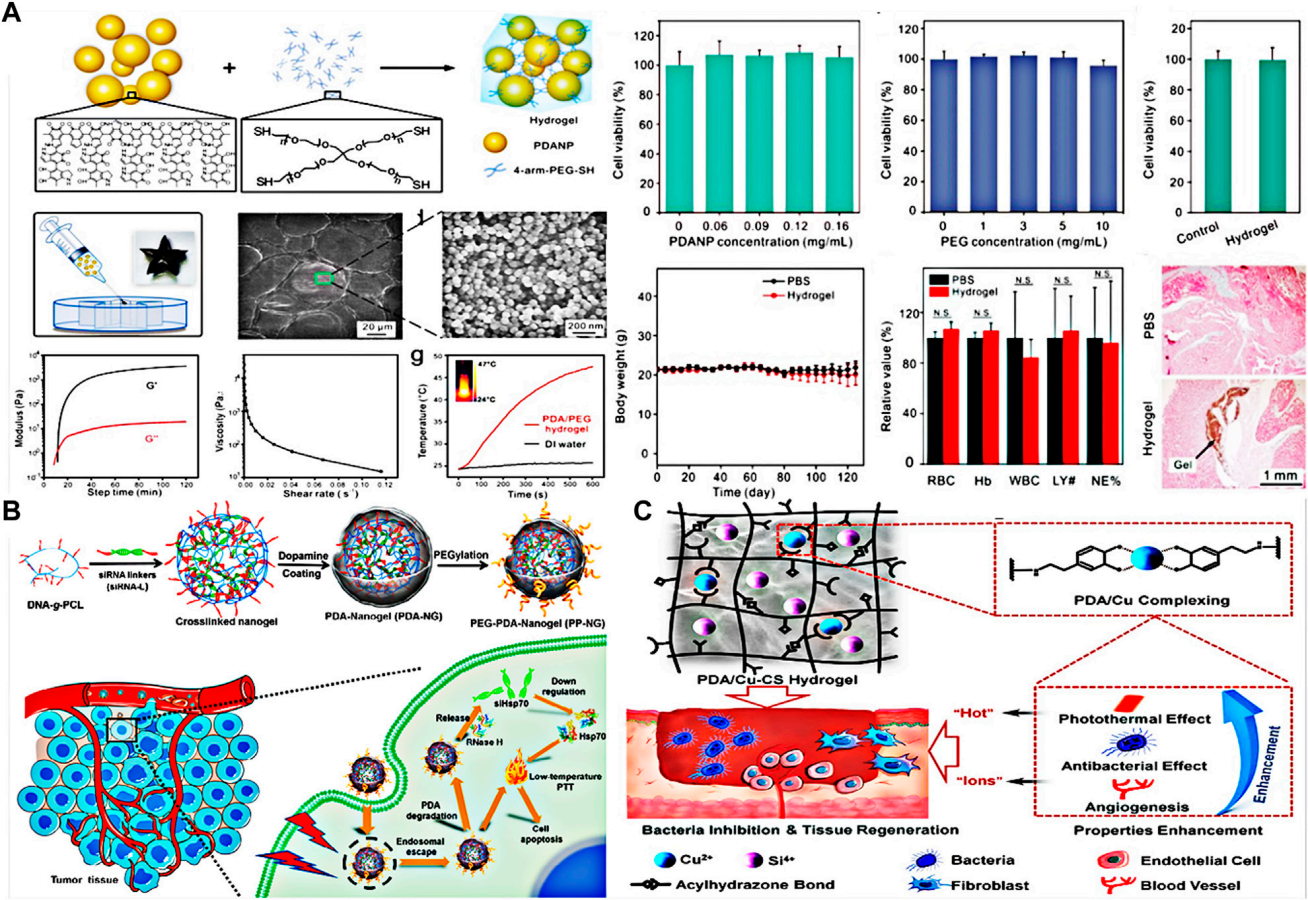
**(A)** Preparation and performance test of PDA-PEG hybrid hydrogels. Reproduced from ref. 80 with permission from 2017, American Chemical Society. **(B)** Preparation of polydopamine coated nucleic acid hydrogels. Reproduced from ref. 15 with permission from 2020 Elsevier Ltd. **(C)** Preparation of polydopamine combined Cu-CS hydrogel and its application in antibacterial. Reproduced from ref. 92 with permission from 2020, American Chemical Society.

Dopamine (DA) is a biocompatible neurotransmitter in human body. It can synthesize polydopamine (PDA) by oxidative self-polymerization, and has different photothermal properties. Biologically inspired poly (dopamine) (PDA) based hydrogels have attracted great attention because of their well-known adhesion and biocompatibility (Han et al., 2017; Zhou D. et al., 2020). Wang et al. described a polydopamine nanoparticle conjugated polyethylene glycol hydrogel that could be used for on-demand drug delivery and combined chemotherapy-photothermal therapy under near-infrared irradiation (Figure 6A) (Wang et al., 2017). Most importantly, the hydrogel had good biocompatibility and would not cause inflammation *in vivo*, and the hydrogel-mediated chemophotothermal therapy could effectively inhibit tumor growth. Zheng et al. designed a new injectable thermosensitive nano hydrogel by loading PDA NP and chemotherapy drugs (Zheng et al., 2020). The hydrogel has anti protein adsorption and photothermal effects, and the injectable amphoteric ion thermosensitive hydrogel has the advantage of low pollution. Ding et al. designed a nucleic acid nanogel coated with polydopamine (PDA) (Figure 6B) (Ding et al., 2020). After being coated with a layer of polydopamine, the nanogel not only protects the nanogel from enzymatic degradation, but also enables the nanogel to have good photothermal conversion ability under near-infrared (NIR) light irradiation. The study shows that the surface temperature of medical implants coated with PDA can be increased under NIR irradiation, which can effectively kill the adhering microorganisms on the implant surface. In addition, Xu et al. synthesized multifunctional composite hydrogels with PDA and Cu-doped calcium silicate ceramics (Cu-CS) as the main components(Figure 6C) (Xu Q. et al., 2020). Copper doped calcium silicate bioceramics have unique biological activity. The composition of PDA and Cu-CS enhanced the antibacterial performance through the “thermionic effect” of copper ions and photothermal materials synergetic antibacterial function.

### Organic dye nanomaterials

Organic dye nanomaterials are also a common photothermal nanomaterials. Indocyanine green (ICG) is a water-soluble anionic tricarbocyanine dye with NIR absorption properties of 808 nm laser irradiation (Lee & Chang, 2017; Ma et al., 2019). Because of its low toxicity, it is widely used. In one study, Pan et al. prepared an ICG alginate gel with good photothermal treatment and good biocompatibility. Most importantly, hydrogels have a strong ICG fixation ability, which facilitates the accumulation of photothermal agents (Pan et al., 2019). This fixation can also reduce the potential side effects of ICG spread to surrounding tissues and improve biocompatibility.

Prussian blue (PB) is also a common organic dye nanomaterial. It was called a pigment in history. PB can be prepared in colloidal form by direct synthesis method. It has a strong charge transfer centered at∼700 nm and a large tail in the near-infrared range. The radiation of this band will lead to thermal relaxation, and local hyperthermia can be generated by irradiating in the so-called bio transparent near-infrared window. PB nanoparticles have complete biocompatibility (PB has been approved by FDA) and biodegradability. Fu et al. established an injectable hydrogel containing Prussian blue nanospheres for cancer photothermal therapy (Fu J. et al., 2019). The hydrogel showed satisfactory serum stability and photothermal conversion ability. In addition, the hydrogel containing the photosensitizer nanospheres has better photothermal conversion efficiency than the nanospheres.

Biliflavin is a dark green bile pigment that is a by-product of the breakdown of hemoglobin. In recent years, the endogenous metabolite biliverin has been shown to have high photothermal conversion properties, as well as cell-protective effects with antioxidant and anti-inflammatory properties. Yao et al. designed a bioinspired green hydrogel (BVSF) (Yao et al., 2020). They incorporated biliverdin into a naturally derived silk fibroin matrix and the resultant hydrogel could be used for anti-glioma, photothermal therapy and wound healing. In the presence of biligreen, the temperature of the hydrogels can rapidly increase to higher than 45°C under NIR irradiation. Meanwhile, BVSF hydrogels can stimulate cell proliferation, migration and adhesion, and perform anti-inflammatory properties, and significantly accelerate wound repair and regeneration.

### Composite nanomaterials

**FIGURE 7:**
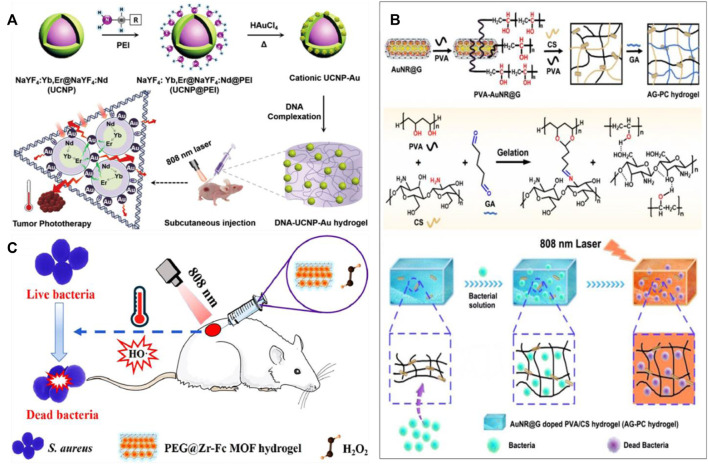
**(A)** Preparation and application of DNA-inorganic hybrid hydrogels. Reproduced from ref. 47 with permission from 2020 Wiley-VCH GmbH. **(B)** Schematic diagram of the procedure for preparation of AG-PC hydrogel and the suggested mechanism and its application in antimicrobial field. Reproduced from ref. 91 with permission from 2019 Royal Society of Chemistry. **(C)** Diagram of the action mechanism of hybrid hydrogels. Reproduced from ref. 79 with permission from Acta Materialia Inc. Published by Elsevier Ltd.

During the construction of hydrogel, in addition to the single photosensitive material, two kinds of composite hybrid materials may play a better effect. Liu et al. prepared a hybrid hydrogel by electrostatic complexation of DNA with upconverted rare-earth Au hybrid nanoparticles (Figure 7A) (Liu et al., 2020). The hybrid hydrogel had a higher photothermal efficiency (42.7%) due to the network formed between DNA and rare-earth Au hybrid nanoparticles. Local administration under 808 nm laser irradiation can achieve tumor eradication without recurrence. Xu and his colleagues prepared AG-PC hybrid hydrogels without antibiotics (Figure 7B) (Xu M.-L. et al., 2019). The hydrogel prepared by doping polyvinyl alcohol (PVA)/chitosan (CS) is highly stable. Because AuNR@G has the photothermal conversion characteristics. Therefore, the hybridized hydrogel showed a highly effective inhibition against both gram-negative *Escherichia coli* and gram-positive *Staphylococcus aureus* under 808 nm laser irradiation. Xing and his colleagues synthesized a collagen hydrogel using self-assembly initiated by gold biomineralization (Xing et al., 2016). Due to the reversible weak interaction between collagen chains and gold nanoparticles, the hydrogel has shear thinning and self-healing functions. This hybrid hydrogel of gold nanoparticles and collagen chains can be used in local drug delivery and sustained release, and provides novel strategy for a wide range of biomedical applications such as drug delivery and tissue engineering. Wang and colleagues synthesized a carrageenan based hybrid hydrogel functionalized with ZR-Fc MOF nanosheets using COOH-PEG-COOH as a carrier (Figure 7C) (Wang X. et al., 2021). The hybrid hydrogel can trap Gram-negative and Gram-positive bacteria by destroying ROS. The hybrid hydrogel can synergistically kill bacteria by decomposing H_2_O_2_ into toxic hydroxyl radicals and photothermal effects.

### Other nanomaterials

**FIGURE 8:**
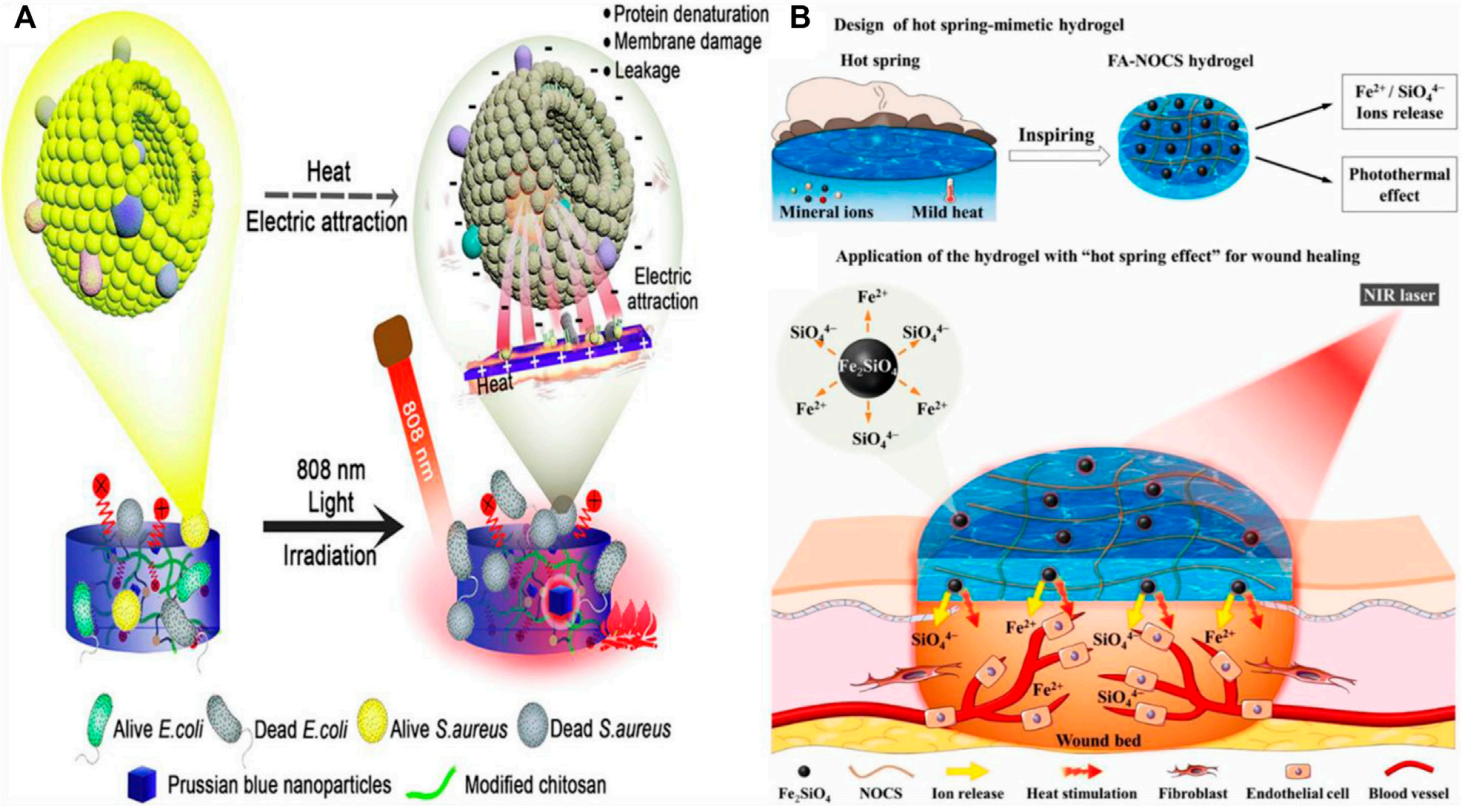
**(A)** Schematic of bactericidal action of hydrogel. Reproduced from ref. 26 with permission from 2020 Elsevier B.V. **(B)** Schematic diagram of application of bioactive photothermal hydrogel in wound healing. Reproduced from ref. 71 with permission from 2020 Elsevier Ltd.

In addition to the above eight photothermal nano-hydrogel materials, there are some other photothermal nano-materials for hydrogel preparation. Ma et al. synthesized a multifunctional Nd Ca Si silicate glass and glass/alginate composite hydrogel (Ma et al., 2020). The hydrogel has fluorescence temperature monitoring performance. Most importantly, due to the addition of bioactive silicate components, the hydrogel has the ability to repair the thermal damage caused by PTT. Therefore, the hydrogel can not only obtain the appropriate PTT temperature for effective treatment of tumors, but also minimize the damage to normal tissues. Han and his colleagues synthesized a new type of photosensitive antibacterial hydrogel (Figure 8A) (Han et al., 2020). The hydrogel can capture bacteria by electrostatic adsorption, and then kill a large number of adsorbed bacteria by high temperature generated by Russel blue MOF particles under near-infrared light. The inhibition rate of *Staphylococcus aureus* and *Escherichia coli* could reach 99.97% and 99.93%, respectively. Sheng and his colleagues synthesized a novel bioactive photothermal nanohybrid hydrogel using Fe-bauxite (Fe_2_SiO_4_) bioceramics and N, O-carboxymethyl chitosan as matrix (Figure 8B) (Sheng et al., 2021). The photothermal nanohybrid hydrogel has good Fe^2+^/SiO_4_^4-^ release and photothermal properties, which can simulate the therapeutic effect of hot spring. Animal Experiments have proved that hydrogels can promote angiogenesis and have great application potential in the field of wound repair materials.

## Biomedical applications of photothermal nanocomposite hydrogels

The application of photothermal nanocomposite hydrogel in biology mainly depends on the photothermal effect of hydrogel itself and the special role of drug loading. Photothermal nanocomposite hydrogels can kill bacteria, inhibit tumor and control drug release through photothermal effect. Drugs released through the photothermal effect can further enhance the killing effect on bacteria and tumors. In addition, the photothermal nanocomposite hydrogel can also enhance the repair of bone tissue. These are described in detail below.

### Photothermal-controlled drug delivery

**FIGURE 9:**
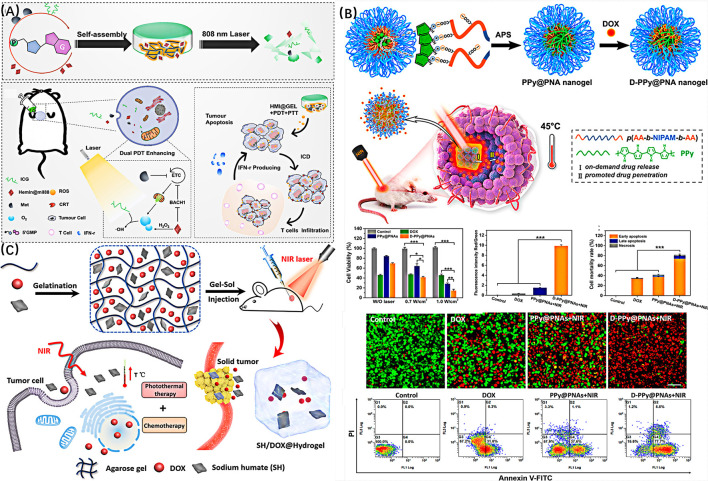
**(A)** Schematic illustration of HMI@GEL. Reproduced from ref. 72 with permission from 2022 Elsevier Ltd. **(B)** Schematic diagram of preparation of temperature sensitive nano hydrogel and its application in temperature controlled drug release for tumor treatment. Reproduced from ref. 22 with permission from 2020, American Chemical Society. **(C)** Schematic diagram of the principle of SH/DOX @ hydrogel controlling drug release and tumor ablation by using photothermal effect. Reproduced from ref. 31 with permission from 2018, American Chemical Society.

One of the main applications of photothermal nanohydrogels is to control the release of drugs by their photothermal properties (Liu C. et al., 2019; Dong et al., 2021). The synergistic treatment of light and heat promotes drugs has better therapeutic effect on diseases. Sun and colleagues combined 5 ′-guanosine monophosphoric acid, indocyanine green, hemin, and metformin to construct a hydrogel HMI@GEL for breast cancer treatment (Figure 9A) (Sun et al., 2022). Due to the photothermal effect of ICG, the hydrogel has good NIR photo-triggering and continuous drug delivery characteristics. Most importantly, the loading concentration of metformin on the hydrogel was as high as 300 mg ml^−1^. This is the highest reported in the literature. The combination of metformin and catalase mimic Hemin@mil88 can not only significantly inhibit mitochondrial respiration in tumors, but also achieve high oxygen production *in situ*. The hydrogel successfully achieves the synchronization of drug synergistic therapy and photo-controlled release under 808 nm laser irradiation, which provides a more reliable direction for the treatment of breast cancer. Zheng and his colleagues prepared a temperature sensitive injectable hydrogel of poly (N-isopropylacrylamide-co-sulfonamide methacrylate) (PNS) in the zwitterionic structure (Zheng et al., 2020). The aqueous dispersion of the nano gel is colloidal at room temperature, and the hydrogel is formed due to thermal sensitivity at 36°C. After the chemotherapeutic drug DOX and photothermal agent PDA nanoparticles are loaded on the hydrogel, DOX can be continuously released from the hydrogel, and the drug release can be accelerated by near-infrared laser irradiation. The synergistic effect of photothermal therapy and local chemotherapy shows a better anti-cancer effect. Geng and his colleagues prepared polyacrylic acid-B-N-isopropylamid-B-acrylic acid/polypyrrole the temperature sensitive composite polymer nanogel through redox polymerization in PNA micelles dissolved in pyrrole (PPy@PNA) (Figure 9B) (Geng et al., 2020). The hydrogel has sensitive sol-gel phase transition behavior, shear dilution characteristics and excellent photothermal properties. It can induce drug release through NIR, and promote drug penetration in tumors. Hou et al. synthesized a powerful injectable agarose hydrogel containing sodium humate and doxorubicin (Figure 9C) (Hou et al., 2018). Under near-infrared light irradiation, SH can effectively convert light energy into heat energy, thereby inducing local high temperature, and continuously release drugs through typical gel sol transition. The drug release rate can be controlled by changing the concentration of agarose, SH and DOX, or the laser power density and irradiation time. Animal experiments show that this light triggered drug release and local hyperthermia combined with chemotherapy photothermal therapy have excellent tumor inhibition.

### Photothermal bacterial killing and wound repair

The harm caused by bacterial infection has been puzzling people. Antibiotics can be used for wound healing to avoid bacterial infection. Long term use of antibiotics may lead to drug resistance. The commonly used gold ion antibacterial is reduced because of its toxicity. Therefore, photothermal therapy has been introduced into the field of antibacterial, it provides an effective treatment strategy for wound infection (Xu J.-W. et al., 2019; Chen et al., 2020).

**FIGURE 10:**
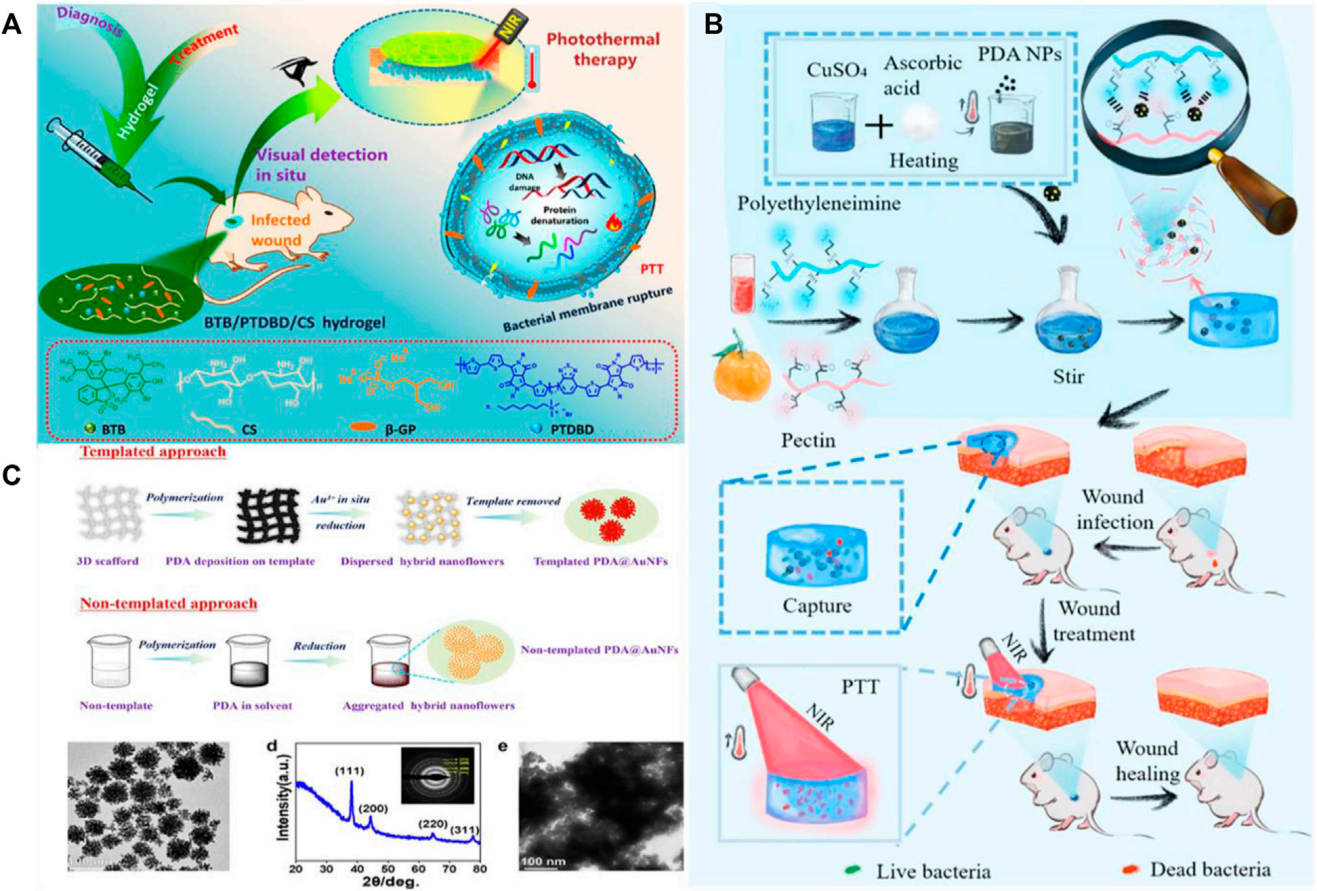
**(A)** Preparation of thermosensitive chitosan hydrogel and its application in the diagnosis and photothermal treatment of bacterial infection. Reproduced from ref. 78 with permission from 2020, American Chemical Society. **(B)** Synthesis and antimicrobial representation of CPAP/PDA@Cu hydrogels. Reproduced from ref. 41 with permission from 2022 The Authors. Published by Elsevier Ltd. **(C)** Preparation diagram and performance characterization of PDA@AuNFs. Reproduced from ref. 30 with permission from 2022 Elsevier B.V.

Wang et al. combined pH sensitive bromothymol blue and near-infrared absorption conjugated polymer into heat sensitive chitosan hydrogel (Figure 10A) (Wang et al., 2020b). Diagnose of the biofilm of *Staphylococcus aureus* (*Staphylococcus aureus*) and the acidic microenvironment of infected wounds were carried out by visible color changes in hydrogels. After rapid diagnosis, hydrogels can be used to treat infect sites, even stubborn biofilms that are difficult to eradicate, by hyperthermia under the irradiation of NIR laser (808 nm). Through thermotherapy, it has broad-spectrum antibacterial activity against gram positive, gram negative and drug-resistant bacteria. Han and his colleagues prepared a GelDA/PGO hydrogel through dopamine grafted gelatin (GelDA) and polydopamine coated graphene oxide (PGO). The introduction of graphene oxide makes hydrogels have excellent photothermal antibacterial properties and is beneficial to enhance wound healing *in vivo* (Han et al., 2022). Deng et al. put single fatty acid Fe (III) (TA Fe) nanoparticles in agarose (AG) hydrogel (Deng H. et al., 2020). When the NIR was irradiated for 10 min, the temperature sharply increased to 58°C, indicating that the nanocomposite hydrogel produced had significant photothermal effects. Through *in vitro* antibacterial test, the hydrogel can effectively kill nearly 99% of bacteria under 10min NIR irradiation. Li and his colleagues prepared a hydrogel with photothermal properties by *in-situ* culturing Cu NPs on the surface of polydopamine and introducing an electrolyte hydrogel precursor (Figure 10B) (Li Z. et al., 2022). Its photothermal properties are better than those of pure polydopamine nanoparticles, and it also can capture and kill bacteria through electrostatic adsorption, which helps to improve the antibacterial performance. In addition, You and others also put forward their own views. They prepared a multifunctional hydrogel wound dressing using copper/tannic acid nanosheets (You et al., 2022). In addition to absorption exudate, the dressing has adjustable photothermal antibacterial and reactive oxygen species scavenging properties. These properties can not only play the role of hemostasis, antibacterial and anti-inflammatory, but also achieve wound repair and restore skin physiological function by reducing inflammation. Hong et al. selected the bacterial cellulose scaffold as the template platform for polydopamine deposition, controlled the growth of mixed polydopamine and gold nanoparticles through *in situ* deposition and reduction technology, and controlled the template platform within 100 nm (Figure 10C) (Hong et al., 2022). Under the irradiation of NIR, the template showed good photothermal performance, and the photothermal temperature could rise from 45°C to 55°C, with good antibacterial effect. Yin et al. used the photothermal properties of copper disulfide nanoparticles to prepare hydrogels through metal coordination (Yin et al., 2022). The photothermal antibacterial efficiency of hydrogels against *Staphylococcus aureus* and *Escherichia coli* can reach 99%. At the same time, it can reduce inflammation and promote skin tissue.

### Photothermal cancer cell inhibition

In today’s society, tumors threaten people’s life and health, but the effect of traditional surgical resection and chemotherapy is not very ideal. People began to care about other effective treatments to treat tumors. The application of photothermal nanomaterials in disease therapy has attracted great attention (Ruhi et al., 2018; Yan et al., 2022). Yang and his colleagues developed a methylcellulose hydrogel platform with photothermal properties and injectable properties (Yang et al., 2021). The hydrogel can be rapidly heated to more than 50.0°C under near-infrared irradiation to achieve the goal of killing tumor cells and preventing tumor recurrence after surgery *in vivo*. The addition of MP in hydrogels can not only improve the strength of hydrogels, but also facilitate the attachment of normal breast cells and adipocytes to promote breast reconstruction. Liu et al. developed a bio-inorganic hybrid hydrogel with near-infrared light response (Liu et al., 2020). The addition of DNA in the NIR response system makes the hydrogel a porous interconnected structure. The interaction between adjacent DNA strands and UCNP-Au nanoparticles makes the photothermal efficiency of the hydrogel as high as 42.7%, and the tumor can be eradicated under 808 nm laser irradiation. Zhou and his colleagues reported an injectable self-healing hydrogel system based on CuS nanoparticles (Zhou et al., 2021). Hydrogels were constructed by forming a 3D network of polyethylene glycol functionalized CuS nanoparticles with surface amino groups with oxidized dextran and PEG with amino terminal groups. The introduction of CuS NPs endows hydrogels with excellent photothermal properties and can inhibit tumor growth in a subcutaneous skin-tumor model. Interestingly, the hydrogel also continuously releases Cu^2+^, which can promote the proliferation of fibroblasts and vascular endothelial cells. Lee and his colleagues synthesized a biodegradable hemoglobin hydrogel (Lee et al., 2019). The hydrogel was constructed by the rapid formation of PEG linkage between hemoglobin and polyethylene glycol *in situ*. The hemoglobin hydrogel was heated to 60°C under near-infrared laser irradiation, which could effectively inhibit A549 lung cancer cells. Most importantly, the hemoglobin has good biocompatibility and can be completely degraded in 21 days after implantation.

### Photothermal-enhanced bone tissue regeneration

**FIGURE 11:**
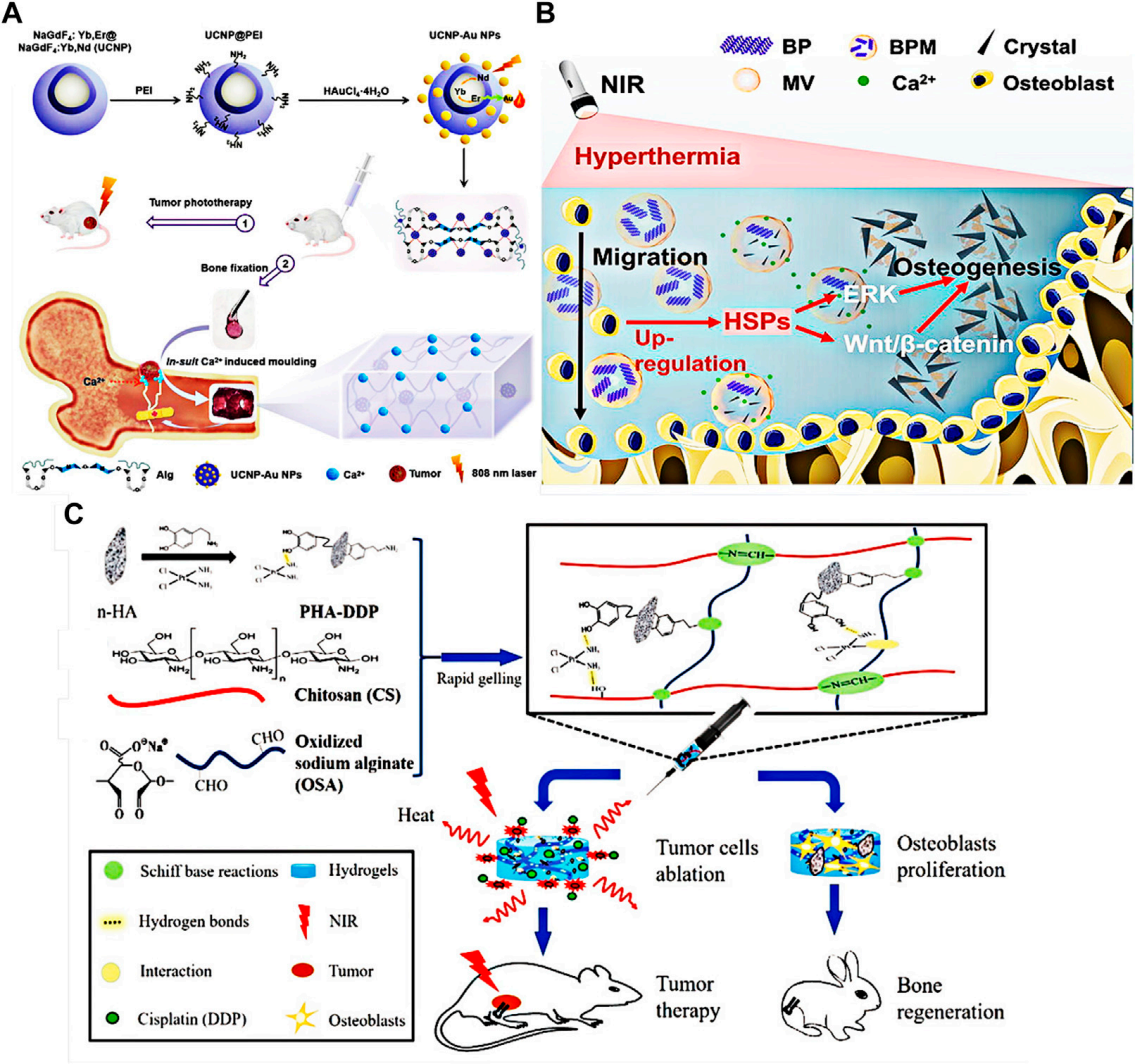
**(A)** Schematic diagram of preparation and principle of near-infrared hydrogel that can promote bone healing. Reproduced from ref. 46 with permission from 2021 Wiley-VCH GmbH. **(B)** Schematic diagram of the principle of photothermal nanohydrogel promoting bone regeneration. Reproduced from ref. 74 with permission from 2021 Elsevier B.V. **(C)** Preparation of cisplatin and dopamine-modified nanohydroxyapatite hydrogel and its application in cancer therapy. Reproduced from ref. 53 with permission from 2019 WILEY-VCH.

The number of patients with bone defects and osteoarthritis is increasing. It not only brings pain to patients, but also is a major problem in clinical treatment. The main reason for the failure of bone defect and osteoarthritis repair is the loss of osteoblasts and chondrocytes. (Marchev et al., 2017). The biomineralization of calcium and phosphorus ions in extracellular matrix is the key to bone regeneration. (de Melo Pereira & Habibovic, 2018; Cheng et al., 2020). At present, many scholars have introduced photothermal therapy into orthopedic repair, providing a new strategy for this field (Wang et al., 2020a; Chang et al., 2022). Wu et al. prepared hydrogels by using the photothermal properties of polydopamine nanoparticles and methacryloyl gelatin (Wu et al., 2022). Animal studies have shown that, hydrogels can promote the alkaline ALP activity and the formation of extracellular calcified nodules. Polydopamine nanoparticles can provide mild photothermal treatment and have better bone repair ability. Liu et al. synthesized a new NIR hydrogel with high photoresponse and mechanical strength using rare-earth gold hybrid nanoparticles and alginate molecules (Figure 11A) (Liu et al., 2021). The hydrogel can not only eradicate tumors by local photothermal therapy, but also effectively promote bone repair as an internal matrix.

Miao and his colleagues prepared nanocomposite hydrogels through BP nano sheets (Miao et al., 2019). Under near-infrared radiation, nanocomposite hydrogels have effective photothermal antibacterial properties. In the absence of bone induction factors, hydrogel matrix could enhance mineralization and bone regeneration, and promote bone formation *in vitro*. Tan et al. prepared EMC simulated hydrogel through BP nano sheet coated by MSC membrane (Figure 11B) (Tan et al., 2022). Under NIR irradiation, they activated heat shock proteins mediated matrix metalloproteinases (MMPs) to induce mild photothermal effect and stimulate the recruitment of osteoblasts. At the same time, the thermal decomposition of BP will release phosphate ions into the surrounding medium and attract calcium ions to form hydroxyapatite in the ECM, which is conducive to the migration and differentiation of osteoblasts and achieves the effect of bone repair.

In addition, Qing et al. added MgO nanoparticles and black phosphorus nanoparticles into poly (vinyl alcohol)/chitosan hydrogels (Qing et al., 2022). The hydrogel can kill more than 99.9% of *Staphylococcus aureus* and *Escherichia coli* under NIR irradiation. The released Mg ions stimulate mesenchymal stem cells to migrate to the hydrogel, and cooperate with the released phosphate to promote osteogenic differentiation, then synergistic photothermal antibacterial and bone regeneration can be achieved. Luo et al. successfully synthesized hydrogels containing cisplatin and dopamine-modified nanohydroxyapatite by Schiff base reaction between aldehyde group on sodium alginate and amino group on chitosan (Figure 11C) (Luo et al., 2019). The results show that the photothermal properties of hydrogels under near-infrared laser (808 nm) irradiation can effectively ablate tumor cells *in vitro* and inhibit tumor growth *in vivo*. Most importantly, because of the abundant functional groups on dopamine, hydrogels can also promote the adhesion and proliferation of bone marrow mesenchymal stem cells, further promote the formation of bone tissue.

### Other biomedical applications

The eye is an important and special organ of the human body, with unique physiological and anatomical characteristics. If eye diseases occur, it is difficult to cure. With the frequent use of lighting screens, eye diseases have become an increasingly serious phenomenon (Li et al., 2021). Although there are many drugs for relieving or treating eye in drops, powders and ointments on the market, there are many deficiencies in their use, such as low permeability, low bioavailability, short stay time, frequent administration, etc. The intraocular bioavailability of these drugs is very low, usually only 1–5% (Wang F. et al., 2021). At present, some non-traditional ocular drug delivery systems have been extensively studied, such as nano carriers, hydrogels, liposomes, etc. Researchers began to introduce photothermal nano hydrogels into ocular drug delivery systems. Pang et al. developed a mini eye patch based on photothermal conversion hydrogel (Pang et al., 2019). The hydrogel eye piece was prepared by cross-linking gelatin and gold nanoparticles. The heating performance of eye piece was obtained through infrared temperature profile and cycling temperature experiments. The results show that the eye system can perceive a variety of visible light and react through spontaneous heating. Through the hydrogel patch, it can convert all kinds of light into heat, stimulate the lacrimal gland to produce more tears to alleviate dry eye.

Microfluidics refers to the science and technology involved in the system of using micro pipes (tens to hundreds of microns in size) to process or manipulate micro fluids (nano liters to a liter in volume). Through electrical stimulation to regulate and transfer plasma nanomaterials, photosensitive materials are introduced to prepare a hydrogel based microfluidic platform with photothermal response, which can effectively provide photothermal therapy in tumor treatment. Ha and his colleagues developed one microfluidic platform based on electric response hydrogel for brain tumor targeting and photothermal therapy (Ha et al., 2020). Electroresponsive hydrogels are composed of silver nanowires (Ag NWs) with high conductivity and biocompatible collagen type I gels. The electroresponsive hydrogel based microfluidic actuator platform can deliver the electroresponsive smart nanomaterials, while the vasopeptide coupled gold nanorods provide photothermal therapy. The combination of electric response and photothermal therapy can promote the release of tumor drugs and effectively improve the therapeutic effect. Fu et al. introduced the principle of photothermal sensor into the analysis device based on microfluidic paper (μ PADs), a photothermal microfluidic sensing platform with multi-channel dual-mode quantitative readout driven by a NIR laser is developed (Fu G. et al., 2019). Prussian blue was used as an analyte related photothermal agent, which was synthesized *in situ* in the thermal reaction poly (n-isopropylacrylamide) hydrogel as a photothermal sensor on the chip. The photothermal effect driven by the NIR laser not only triggered the dose dependent heat generation on the chip, but also triggered the phase change induced release of hydrogel dye, and enabled the dual-mode vision based on thermal image and distance to read the analyte concentration quantitatively in a multi-channel manner. The elevated temperature of the hydrogel on the tablet and the moving distance of the dye solution released are directly proportional to the concentration of PB.

## Conclusion and perspectives

The application and development of metal nanomaterials, carbon-based nanomaterials, metal sulfide/oxide nanoparticles, black phosphorus nanomaterials, MXenes nanomaterials, polymer nanomaterials and organic dye materials in the preparation of photothermal nano hydrogels are reviewed in this paper. The applications of photothermal nano hydrogels in drug release, photothermal sterilization, photothermal cancer cell inhibition and bone repair enhancement were introduced in detail. Photothermal nano hydrogels can inhibit the growth of bacteria and tumor cells through the high temperature generated by the photothermal effect, and control the sol-gel transition behavior of hydrogels through the photothermal characteristics, thus control the drug release. The synergistic effect of photothermal therapy and chemotherapy can greatly enhance the therapeutic effect and reduce the drug toxicity. There is no doubt that the photothermal therapy of local hyperthermia combined with chemotherapy will have further applications in medical engineering.

## References

[B1] Ai K. Huang J. Xiao Z. Yang Y. Bai Y. Peng J. (2021). Localized surface plasmon resonance properties and biomedical applications of copper selenide nanomaterials. Mater. Today Chem. 20, 100402. 10.1016/j.mtchem.2020.100402

[B2] Amatya R. Hwang S. Park T. Chung Y. J. Ryu S. Lee J. (2021). BSA/Silver nanoparticle-loaded hydrogel film for local photothermal treatment of skin cancer. Pharm. Res. 38, 873–883. 10.1007/s11095-021-03038-4 33835356

[B3] Awasthi P. An X. Xiang J. Kalva N. Shen Y. Li C. (2020). Facile synthesis of noncytotoxic PEGylated dendrimer encapsulated silver sulfide quantum dots for NIR-II biological imaging. Nanoscale 12, 5678–5684. 10.1039/c9nr10918h 32101213

[B4] Bermudez-Jimenez C. Nino-Martinez N. Patino-Marin N. Martinez-Gutierrez F. Ruiz F. Bach H. (2020). Effective control of biofilms by photothermal therapy using a gold nanorod hydrogel. J. Biomed. Mat. Res. 108, 333–342. 10.1002/jbm.b.34392 31041849

[B5] Cao H. Duan L. Zhang Y. Cao J. Zhang K. (2021). Current hydrogel advances in physicochemical and biological response-driven biomedical application diversity. Signal Transduct. Target. Ther. 6, 426–431. 10.1038/s41392-021-00830-x 34916490 PMC8674418

[B6] Chang L. Liu X. Zhu J. Rao Y. Chen D. Wang Y. (2022). Cellulose-based thermo-responsive hydrogel with NIR photothermal enhanced DOX released property for anti-tumor chemotherapy. Colloids Surfaces B Biointerfaces 218, 112747. 10.1016/j.colsurfb.2022.112747 35961115

[B7] Chen S. Tang F. Tang L. Li L. (2017). Synthesis of Cu-nanoparticle hydrogel with self-healing and photothermal properties. ACS Appl. Mat. Interfaces 9, 20895–20903. 10.1021/acsami.7b04956 28569057

[B8] Chen T. Yao T. Peng H. Whittaker A. K. Li Y. Zhu S. (2021). An injectable hydrogel for simultaneous photothermal therapy and photodynamic therapy with ultrahigh efficiency based on carbon dots and modified cellulose nanocrystals. Adv. Funct. Mat. 31, 2106079. 10.1002/adfm.202106079

[B9] Chen Y. Gao Y. Chen Y. Liu L. Mo A. Peng Q. (2020). Nanomaterials-based photothermal therapy and its potentials in antibacterial treatment. J. Control. Release 328, 251–262. 10.1016/j.jconrel.2020.08.055 32889053

[B10] Cheng L. Chen Z. Cai Z. Zhao J. Lu M. Liang J. (2020). Bioinspired functional black phosphorus electrospun fibers achieving recruitment and biomineralization for staged bone regeneration. Small 16, 2005433. 10.1002/smll.202005433 33230977

[B11] de Melo Pereira D. Habibovic P. (2018). Biomineralization‐inspired material design for bone regeneration. Adv. Healthc. Mat. 7, 1800700. 10.1002/adhm.201800700 30240157

[B12] de Oliveira Lima A. M. Fragal E. H. Caldas B. S. Nakamura T. U. Rubira A. F. Silva R. (2022). Functional mesoporous silica decorated with Ag nanoparticles as chemo-photothermal agents. Microporous Mesoporous Mater. 341, 112097. 10.1016/j.micromeso.2022.112097

[B13] Deng H. Yu Z. Chen S. Fei L. Sha Q. Zhou N. (2020a). Facile and eco-friendly fabrication of polysaccharides-based nanocomposite hydrogel for photothermal treatment of wound infection. Carbohydr. Polym. 230, 115565. 10.1016/j.carbpol.2019.115565 31887966

[B14] Deng X. Liang H. Yang W. Shao Z. (2020b). Polarization and function of tumor-associated macrophages mediate graphene oxide-induced photothermal cancer therapy. J. Photochem. Photobiol. B Biol. 208, 111913. 10.1016/j.jphotobiol.2020.111913 32473533

[B15] Ding F. Gao X. Huang X. Ge H. Xie M. Qian J. (2020). Polydopamine-coated nucleic acid nanogel for siRNA-mediated low-temperature photothermal therapy. Biomaterials 245, 119976. 10.1016/j.biomaterials.2020.119976 32213362

[B16] Dong Y. Li S. Li X. Wang X. (2021). Smart MXene/agarose hydrogel with photothermal property for controlled drug release. Int. J. Biol. Macromol. 190, 693–699. 10.1016/j.ijbiomac.2021.09.037 34520776

[B17] Eswaraiah V. Zeng Q. Long Y. Liu Z. (2016). Black phosphorus nanosheets: Synthesis, characterization and applications. Small 12, 3480–3502. 10.1002/smll.201600032 27225670

[B18] Falke Y. Senkovskiy B. V. Ehlen N. Wysocki L. Marangoni T. Durr R. A. (2020). Photothermal bottom-up graphene nanoribbon growth kinetics. Nano Lett. 20, 4761–4767. 10.1021/acs.nanolett.0c00317 32510961

[B19] Fu G. Zhu Y. Xu K. Wang W. Hou R. Li X. (2019a). Photothermal microfluidic sensing platform using near-infrared laser-driven multiplexed dual-mode visual quantitative readout. Anal. Chem. 91, 13290–13296. 10.1021/acs.analchem.9b04059 31508942

[B20] Fu J. J. Zhang J. Y. Li S. P. Zhang L. M. Lin Z. X. Liang L. (2018). CuS nanodot-loaded thermosensitive hydrogel for anticancer photothermal therapy. Mol. Pharm. 15, 4621–4631. 10.1021/acs.molpharmaceut.8b00624 30179511

[B21] Fu J. Wu B. Wei M. Huang Y. Zhou Y. Zhang Q. (2019b). Prussian blue nanosphere-embedded *in situ* hydrogel for photothermal therapy by peritumoral administration. Acta Pharm. Sin. B 9, 604–614. 10.1016/j.apsb.2018.12.005 31193840 PMC6543023

[B22] Geng S. Zhao H. Zhan G. Zhao Y. Yang X. (2020). Injectable *in situ* forming hydrogels of thermosensitive polypyrrole nanoplatforms for precisely synergistic photothermo-chemotherapy. ACS Appl. Mat. Interfaces 12, 7995–8005. 10.1021/acsami.9b22654 32013384

[B23] Guedes G. Wang S. Fontana F. Figueiredo P. Lindén J. Correia A. (2021). Dual‐crosslinked dynamic hydrogel incorporating {Mo154} with pH and NIR responsiveness for chemo‐photothermal therapy. Adv. Mater. 33, 2007761. 10.1002/adma.202007761 PMC11468987 34382257

[B24] Gupta N. Malviya R. (2021). Understanding and advancement in gold nanoparticle targeted photothermal therapy of cancer. Biochimica Biophysica Acta - Rev. Cancer 1875, 188532. 10.1016/j.bbcan.2021.188532 33667572

[B25] Ha J. H. Shin H. H. Choi H. W. Lim J. H. Mo S. J. Ahrberg C. D. (2020). Electro-responsive hydrogel-based microfluidic actuator platform for photothermal therapy. Lab. Chip 20, 3354–3364. 10.1039/d0lc00458h 32749424

[B26] Han D. Li Y. Liu X. Li B. Han Y. Zheng Y. (2020). Rapid bacteria trapping and killing of metal-organic frameworks strengthened photo-responsive hydrogel for rapid tissue repair of bacterial infected wounds. Chem. Eng. J. 396, 125194. 10.1016/j.cej.2020.125194

[B27] Han K. Bai Q. Zeng Q. Sun N. Zheng C. Wu W. (2022). A multifunctional mussel-inspired hydrogel with antioxidant, electrical conductivity and photothermal activity loaded with mupirocin for burn healing. Mater. Des. 217, 110598. 10.1016/j.matdes.2022.110598

[B28] Han L. Lu X. Liu K. Wang K. Fang L. Weng L.-T. (2017). Mussel-inspired adhesive and tough hydrogel based on nanoclay confined dopamine polymerization. ACS Nano 11, 2561–2574. 10.1021/acsnano.6b05318 28245107

[B29] He P. P. Du X. Cheng Y. Gao Q. Liu C. Wang X. (2022). Thermal‐responsive MXene‐DNA hydrogel for near‐infrared light triggered localized photothermal‐chemo synergistic cancer therapy. Small 18, 2200263. 10.1002/smll.202200263 36056901

[B30] Hong J. Chen M. Xian J. Li C. Zheng X. Deng Q. (2022). Preparation of Au-based hybrid nanoflowers as efficient photothermal agents for antibacterial application. Mater. Lett. 317, 132034. 10.1016/j.matlet.2022.132034

[B31] Hou M. Yang R. Zhang L. Zhang L. Liu G. Xu Z. (2018). Injectable and natural humic acid/agarose hybrid hydrogel for localized light-driven photothermal ablation and chemotherapy of cancer. ACS Biomater. Sci. Eng. 4, 4266–4277. 10.1021/acsbiomaterials.8b01147 33418824

[B32] Hou X. L. Dai X. Yang J. Zhang B. Zhao D. H. Li C. Q. (2020). Injectable polypeptide-engineered hydrogel depot for amplifying the anti-tumor immune effect induced by chemo-photothermal therapy. J. Mat. Chem. B 8, 8623–8633. 10.1039/d0tb01370f 32821893

[B33] Huang F. G. (2020). Nanovehicles with nitric oxide release and photothermal activity-based hydrogels for bacteria-infected wound healing. ACS Appl. Mat. Interfaces 12 (26), 28952–28964. 10.1021/acsami.0c04080 32475108

[B34] Jin R. Yang J. Ding P. Li C. Zhang B. Chen W. (2020). Antitumor immunity triggered by photothermal therapy and photodynamic therapy of a 2D MoS_2_ nanosheet-incorporated injectable polypeptide-engineered hydrogel combinated with chemotherapy for 4T1 breast tumor therapy. Nanotechnology 31, 205102. 10.1088/1361-6528/ab72b9 32018232

[B35] Lee C. Lim K. Kim S. S. Lee E. S. Oh K. T. Choi H.-G. (2019). Near infrared light-responsive heat-emitting hemoglobin hydrogels for photothermal cancer therapy. Colloids Surfaces B Biointerfaces 176, 156–166. 10.1016/j.colsurfb.2018.12.070 30611939

[B36] Lee H. Kim S. Ryu C. Lee J. Y. (2020). Photothermal polymerization using graphene oxide for robust hydrogelation with various light sources. ACS Biomater. Sci. Eng. 6, 1931–1939. 10.1021/acsbiomaterials.0c00161 33455321

[B37] Lee Y.-H. Chang D.-S. (2017). Fabrication, characterization, and biological evaluation of anti-HER2 indocyanine green-doxorubicin-encapsulated PEG-b-PLGA copolymeric nanoparticles for targeted photochemotherapy of breast cancer cells. Sci. Rep. 7, 46688–46713. 10.1038/srep46688 28429764 PMC5399361

[B38] Li Q. Wen J. Liu C. Jia Y. Wu Y. Shan Y. (2019). Graphene-nanoparticle-based self-healing hydrogel in preventing postoperative recurrence of breast cancer. ACS Biomater. Sci. Eng. 5, 768–779. 10.1021/acsbiomaterials.8b01475 33405838

[B39] Li Y. Han M. Cai Y. Jiang B. Zhang Y. Yuan B. (2022a). Muscle-inspired MXene/PVA hydrogel with high toughness and photothermal therapy for promoting bacteria-infected wound healing. Biomater. Sci. 10, 1068–1082. 10.1039/d1bm01604k 35037673

[B40] Li Z. Cheng H. Ke L. Liu M. Wang C. G. Jun Loh X. (2021). Recent advances in new copolymer hydrogel‐formed contact lenses for ophthalmic drug delivery. ChemNanoMat 7, 564–579. 10.1002/cnma.202100008

[B41] Li Z. You S. Mao R. Xiang Y. Cai E. Deng H. (2022b). Architecting polyelectrolyte hydrogels with Cu-assisted polydopamine nanoparticles for photothermal antibacterial therapy. Mater. Today Bio 15, 100264. 10.1016/j.mtbio.2022.100264 PMC9062430 35517578

[B42] Liang Y. Zhao X. Hu T. Chen B. Yin Z. Ma P. X. (2019). Adhesive hemostatic conducting injectable composite hydrogels with sustained drug release and photothermal antibacterial activity to promote full-thickness skin regeneration during wound healing. Small 15, e1900046. 10.1002/smll.201900046 30786150

[B43] Lin H. Chen Y. Shi J. (2018). Insights into 2D MXenes for versatile biomedical applications: Current advances and challenges ahead. Adv. Sci. (Weinh). 5, 1800518. 10.1002/advs.201800518 30356929 PMC6193163

[B44] Lin H. Wang X. Yu L. Chen Y. Shi J. (2017). Two-dimensional ultrathin MXene ceramic nanosheets for photothermal conversion. Nano Lett. 17, 384–391. 10.1021/acs.nanolett.6b04339 28026960

[B45] Lin X. Fang Y. Hao Z. Wu H. Zhao M. Wang S. (2021). Bacteria-triggered multifunctional hydrogel for localized chemodynamic and low-temperature photothermal sterilization. Small 17, e2103303. 10.1002/smll.202103303 34643054

[B46] Liu B. Gu X. Sun Q. Jiang S. Sun J. Liu K. (2021). Injectable *in situ* induced robust hydrogel for photothermal therapy and bone fracture repair. Adv. Funct. Mat. 31, 2010779. 10.1002/adfm.202010779

[B47] Liu B. Sun J. Zhu J. Li B. Ma C. Gu X. (2020). Injectable and NIR‐responsive DNA–inorganic hybrid hydrogels with outstanding photothermal therapy. Adv. Mat. 32, 2004460. 10.1002/adma.202004460 32830376

[B48] Liu C. Guo X. Ruan C. Hu H. Jiang B.-P. Liang H. (2019a). An injectable thermosensitive photothermal-network hydrogel for near-infrared-triggered drug delivery and synergistic photothermal-chemotherapy. Acta biomater. 96, 281–294. 10.1016/j.actbio.2019.07.024 31319202

[B49] Liu M. Huang P. Wang W. Feng Z. Zhang J. Deng L. (2019b). An injectable nanocomposite hydrogel co-constructed with gold nanorods and paclitaxel-loaded nanoparticles for local chemo-photothermal synergetic cancer therapy. J. Mat. Chem. B 7, 2667–2677. 10.1039/c9tb00120d 32255000

[B50] Liu W. Zhang X. Zhou L. Shang L. Su Z. (2019c). Reduced graphene oxide (rGO) hybridized hydrogel as a near-infrared (NIR)/pH dual-responsive platform for combined chemo-photothermal therapy. J. Colloid Interface Sci. 536, 160–170. 10.1016/j.jcis.2018.10.050 30366181

[B51] Lu X. Hou J. Yang K. Zhu L. Xing B. Lin D. (2021a). Binding force and site-determined desorption and fragmentation of antibiotic resistance genes from metallic nanomaterials. Environ. Sci. Technol. 55, 9305–9316. 10.1021/acs.est.1c02047 34138538

[B52] Lu Y. Fan D. Wang Y. Xu H. Lu C. Yang X. (2021b). Surface patterning of two-dimensional nanostructure-embedded photothermal hydrogels for high-yield solar steam generation. ACS Nano 15, 10366–10376. 10.1021/acsnano.1c02578 34110789

[B53] Luo S. Wu J. Jia Z. Tang P. Sheng J. Xie C. (2019). An injectable, bifunctional hydrogel with photothermal effects for tumor therapy and bone regeneration. Macromol. Biosci. 19, 1900047. 10.1002/mabi.201900047 31318163

[B54] Ma H. Zhou Q. Chang J. Wu C. (2019). Grape seed-inspired smart hydrogel scaffolds for melanoma therapy and wound healing. ACS Nano 13, 4302–4311. 10.1021/acsnano.8b09496 30925040

[B55] Ma L. Zhou Y. Zhang Z. Liu Y. Zhai D. Zhuang H. (2020). Multifunctional bioactive Nd-Ca-Si glasses for fluorescence thermometry, photothermal therapy, and burn tissue repair. Sci. Adv. 6, eabb1311. 10.1126/sciadv.abb1311 32821831 PMC7413731

[B56] Marchev A. S. Dimitrova P. A. Burns A. J. Kostov R. V. Dinkova‐Kostova A. T. Georgiev M. I. (2017). Oxidative stress and chronic inflammation in osteoarthritis: Can NRF2 counteract these partners in crime? Ann. N. Y. Acad. Sci. 1401, 114–135. 10.1111/nyas.13407 28662306

[B57] Miao Y. Shi X. Li Q. Hao L. Liu L. Liu X. (2019). Engineering natural matrices with black phosphorus nanosheets to generate multi-functional therapeutic nanocomposite hydrogels. Biomater. Sci. 7, 4046–4059. 10.1039/c9bm01072f 31435628

[B58] Moorcroft S. C. T. Roach L. Jayne D. G. Ong Z. Y. Evans S. D. (2020). Nanoparticle-loaded hydrogel for the light-activated release and photothermal enhancement of antimicrobial peptides. ACS Appl. Mat. Interfaces 12, 24544–24554. 10.1021/acsami.9b22587 32312040

[B59] Naguib M. Kurtoglu M. Presser V. Lu J. Niu J. Heon M. (2011). Two‐dimensional nanocrystals produced by exfoliation of Ti_3_AlC_2_ . Adv. Mat. 23, 4248–4253. 10.1002/adma.201102306 21861270

[B60] Ou Y. Tian M. (2021). Advances in multifunctional chitosan-based self-healing hydrogels for biomedical applications. J. Mat. Chem. B 9, 7955–7971. 10.1039/d1tb01363g 34611684

[B61] Pan H. Zhang C. Wang T. Chen J. Sun S. K. (2019). *In situ* fabrication of intelligent photothermal indocyanine green-alginate hydrogel for localized tumor ablation. ACS Appl. Mat. Interfaces 11, 2782–2789. 10.1021/acsami.8b16517 30584767

[B62] Pang Y. Wei C. Li R. Wu Y. Liu W. Wang F. (2019). <p&gt;Photothermal conversion hydrogel based mini-eye patch for relieving dry eye with long-term use of the light-emitting screen&lt;/p&gt;. Int. J. Nanomedicine 14, 5125–5133. 10.2147/ijn.s192407 31371951 PMC6628948

[B63] Park H. H. Srisombat L.-o. Jamison A. C. Liu T. Marquez M. D. Park H. (2018). Temperature-responsive hydrogel-coated gold nanoshells. Gels 4, 28. 10.3390/gels4020028 PMC6209258 30674804

[B64] Pei X. Wang J. Cong Y. Fu J. (2021). Recent progress in polymer hydrogel bioadhesives. J. Polym. Sci. 59, 1312–1337. 10.1002/pol.20210249

[B65] Qin L. Ling G. Peng F. Zhang F. Jiang S. He H. (2019). Black phosphorus nanosheets and gemcitabine encapsulated thermo-sensitive hydrogel for synergistic photothermal-chemotherapy. J. Colloid Interface Sci. 556, 232–238. 10.1016/j.jcis.2019.08.058 31446336

[B66] Qing Y. Wang H. Lou Y. Fang X. Li S. Wang X. (2022). Chemotactic ion-releasing hydrogel for synergistic antibacterial and bone regeneration. Mater. Today Chem. 24, 100894. 10.1016/j.mtchem.2022.100894

[B67] Ren Q. Ma Y. Zhang S. Ga L. Ai J. (2021). One-step synthesis of water-soluble silver sulfide quantum dots and their application to bioimaging. ACS omega 6, 6361–6367. 10.1021/acsomega.0c06276 33718726 PMC7948225

[B68] Ren X. Li Z. Huang Z. Sang D. Qiao H. Qi X. (2017). Environmentally robust black phosphorus nanosheets in solution: Application for self‐powered photodetector. Adv. Funct. Mat. 27, 1606834. 10.1002/adfm.201606834

[B69] Ruhi M. K. Ayse A. K. Gülsoy M. (2018). Dose-dependent photochemical/photothermal toxicity of indocyanine green-based therapy on three different cancer cell lines. Photodiagnosis Photodyn. Ther. 21, 334–343. 10.1016/j.pdpdt.2018.01.008 29339061

[B70] Shao J. Ruan C. Xie H. Li Z. Wang H. Chu P. K. (2018). Black-phosphorus-incorporated hydrogel as a sprayable and biodegradable photothermal platform for postsurgical treatment of cancer. Adv. Sci. (Weinh). 5, 1700848. 10.1002/advs.201700848 29876210 PMC5978961

[B71] Sheng L. Zhang Z. Zhang Y. Wang E. Ma B. Xu Q. (2021). A novel “hot spring”-mimetic hydrogel with excellent angiogenic properties for chronic wound healing. Biomaterials 264, 120414. 10.1016/j.biomaterials.2020.120414 32980635

[B72] Sun Y. Fang K. Hu X. Yang J. Jiang Z. Feng L. (2022). NIR-light-controlled G-quadruplex hydrogel for synergistically enhancing photodynamic therapy via sustained delivery of metformin and catalase-like activity in breast cancer. Mater. Today Bio 16, 100375. 10.1016/j.mtbio.2022.100375 PMC9379686 35983175

[B73] Sun Y. Gao J. Liu Y. Kang H. Xie M. Wu F. (2019). Copper sulfide-macroporous polyacrylamide hydrogel for solar steam generation. Chem. Eng. Sci. 207, 516–526. 10.1016/j.ces.2019.06.044

[B74] Tan L. Hu Y. Li M. Zhang Y. Xue C. Chen M. (2022). Remotely-activatable extracellular matrix-mimetic hydrogel promotes physiological bone mineralization for enhanced cranial defect healing. Chem. Eng. J. 431, 133382. 10.1016/j.cej.2021.133382

[B75] Tao B. Lin C. Deng Y. Yuan Z. Shen X. Chen M. (2019). Copper-nanoparticle-embedded hydrogel for killing bacteria and promoting wound healing with photothermal therapy. J. Mat. Chem. B 7, 2534–2548. 10.1039/c8tb03272f 32255130

[B76] Wang F. Song Y. Huang J. Wu B. Wang Y. Pang Y. (2021a). Lollipop‐inspired multilayered drug delivery hydrogel for dual effective, long‐term, and NIR‐defined glaucoma treatment. Macromol. Biosci. 21, 2100202. 10.1002/mabi.202100202 34405963

[B77] Wang H. Zeng X. Pang L. Wang H. Lin B. Deng Z. (2020a). Integrative treatment of anti-tumor/bone repair by combination of MoS_2_ nanosheets with 3D printed bioactive borosilicate glass scaffolds. Chem. Eng. J. 396, 125081. 10.1016/j.cej.2020.125081

[B78] Wang H. Zhou S. Guo L. Wang Y. Feng L. (2020b). Intelligent hybrid hydrogels for rapid *in situ* detection and photothermal therapy of bacterial infection. ACS Appl. Mat. Interfaces 12, 39685–39694. 10.1021/acsami.0c12355 32805886

[B79] Wang X. Sun X. Bu T. Wang Q. Zhang H. Jia P. (2021b). Construction of a photothermal hydrogel platform with two-dimensional PEG@ zirconium-ferrocene MOF nanozymes for rapid tissue repair of bacteria-infected wounds. Acta Biomater. 135, 342–355. 10.1016/j.actbio.2021.08.022 34450338

[B80] Wang X. Wang C. Wang X. Wang Y. Zhang Q. Cheng Y. (2017). A polydopamine nanoparticle-knotted poly(ethylene glycol) hydrogel for on-demand drug delivery and chemo-photothermal therapy. Chem. Mat. 29, 1370–1376. 10.1021/acs.chemmater.6b05192

[B81] Wu C. Zhao J. Hu F. Zheng Y. Yang H. Pan S. (2018). Design of injectable agar-based composite hydrogel for multi-mode tumor therapy. Carbohydr. Polym. 180, 112–121. 10.1016/j.carbpol.2017.10.024 29103486

[B82] Wu R. S. Lin J. Xing Y. M. Dai Z. L. Wang L. W. Zhang X. P. (2019). pH-sensitive black phosphorous-incorporated hydrogel as novel implant for cancer treatment. J. Pharm. Sci. 108, 2542–2551. 10.1016/j.xphs.2019.03.003 30876860

[B83] Wu Y. Liang Y. Liu Y. Hao Y. Tao N. Li J. (2021). A Bi_2_S_3_-embedded gellan gum hydrogel for localized tumor photothermal/antiangiogenic therapy. J. Mat. Chem. B 9, 3224–3234. 10.1039/d1tb00257k 33885626

[B84] Wu Y. Zhang X. Tan B. Shan Y. Zhao X. Liao J. (2022). Near-infrared light control of GelMA/PMMA/PDA hydrogel with mild photothermal therapy for skull regeneration. Biomater. Adv. 133, 112641. 10.1016/j.msec.2022.112641 35034819

[B85] Xie S. Chen Y. Guo Z. Luo Y. Tan H. Xu L. (2021). Agar/carbon dot crosslinked polyacrylamide double-network hydrogels with robustness, self-healing, and stimulus-response fluorescence for smart anti-counterfeiting. Mat. Chem. Front. 5, 5418–5428. 10.1039/d1qm00338k

[B86] Xie Y. Gan C. Li Z. Liu W. Yang D. Qiu X. (2022). Fabrication of a lignin-copper sulfide-incorporated PVA hydrogel with near-infrared-activated photothermal/photodynamic/peroxidase-like performance for combating bacteria and biofilms. ACS Biomater. Sci. Eng. 8, 560–569. 10.1021/acsbiomaterials.1c01406 35077128

[B87] Xing R. Liu K. Jiao T. Zhang N. Ma K. Zhang R. (2016). An injectable self-assembling collagen-gold hybrid hydrogel for combinatorial antitumor photothermal/photodynamic therapy. Adv. Mat. 28, 3669–3676. 10.1002/adma.201600284 26991248

[B88] Xu D. Li Z. Li L. Wang J. (2020a). 2D MXene nanomaterials: Insights into the photothermal conversion of 2D MXene nanomaterials: Synthesis, mechanism, and applications (adv. Funct. Mater. 47/2020). Adv. Funct. Mat. 30, 2070314. 10.1002/adfm.202070314

[B89] Xu H. Shang H. Wang C. Du Y. (2020b). Low‐dimensional metallic nanomaterials for advanced electrocatalysis. Adv. Funct. Mat. 30, 2006317. 10.1002/adfm.202006317

[B90] Xu J.-W. Yao K. Xu Z.-K. (2019a). Nanomaterials with a photothermal effect for antibacterial activities: An overview. Nanoscale 11, 8680–8691. 10.1039/c9nr01833f 31012895

[B91] Xu M.-L. Guan L.-Y. Li S.-K. Chen L. Chen Z. (2019b). Stable gold graphitic nanocapsule doped hydrogels for efficient photothermal antibacterial applications. Chem. Commun. 55, 5359–5362. 10.1039/c9cc01933b 30994651

[B92] Xu Q. Chang M. Zhang Y. Wang E. Xing M. Gao L. (2020c). PDA/Cu bioactive hydrogel with “hot ions effect” for inhibition of drug-resistant bacteria and enhancement of infectious skin wound healing. ACS Appl. Mat. Interfaces 12, 31255–31269. 10.1021/acsami.0c08890 32530593

[B93] Xu T. Liu K. Sheng N. Zhang M. Liu W. Liu H. (2022a). Biopolymer-based hydrogel electrolytes for advanced energy storage/conversion devices: Properties, applications, and perspectives. Energy Storage Mater. 48, 244–262. 10.1016/j.ensm.2022.03.013

[B94] Xu Y. Chen H. Fang Y. Wu J. (2022b2200494). Hydrogel combined with phototherapy in wound healing. Adv. Healthc. Mat. 11, 2200494. 10.1002/adhm.202200494 35751637

[B95] Yan J. Zhang Y. Zheng L. Wu Y. Wang T. Jiang T. (2022). Let‐7i miRNA and platinum loaded nano‐graphene oxide platform for detection/reversion of drug resistance and synergetic chemical‐photothermal inhibition of cancer cell. Chin. Chem. Lett. 33, 767–772. 10.1016/j.cclet.2021.08.018

[B96] Yang X. Gao L. Wei Y. Tan B. Wu Y. Yi C. (2021). Photothermal hydrogel platform for prevention of post-surgical tumor recurrence and improving breast reconstruction. J. Nanobiotechnology 19, 307–313. 10.1186/s12951-021-01041-w 34620160 PMC8499550

[B97] Yao Q. Lan Q. H. Jiang X. Du C. C. Zhai Y. Y. Shen X. (2020). Bioinspired biliverdin/silk fibroin hydrogel for antiglioma photothermal therapy and wound healing. Theranostics 10, 11719–11736. 10.7150/thno.47682 33052243 PMC7545989

[B98] Yin W. Wang Q. Zhang J. Chen X. Wang Y. Jiang Z. (2022). A dynamic nano-coordination protein hydrogel for photothermal treatment and repair of infected skin injury. J. Mat. Chem. B 10, 8181–8185. 10.1039/d2tb01146h 35819200

[B99] You S. Xiang Y. Qi X. Mao R. Cai E. Lan Y. (2022). Harnessing a biopolymer hydrogel reinforced by copper/tannic acid nanosheets for treating bacteria-infected diabetic wounds. Mater. Today Adv. 15, 100271. 10.1016/j.mtadv.2022.100271

[B100] Yu Y.-T. Shi S.-W. Wang Y. Zhang Q.-L. Gao S.-H. Yang S.-P. (2019). A ruthenium nitrosyl-functionalized magnetic nanoplatform with near-infrared light-controlled nitric oxide delivery and photothermal effect for enhanced antitumor and antibacterial therapy. ACS Appl. Mat. Interfaces 12, 312–321. 10.1021/acsami.9b18865 31840976

[B101] Yuan P. Yang T. Liu T. Yu X. Bai Y. Zhang Y. (2020). Nanocomposite hydrogel with NIR/magnet/enzyme multiple responsiveness to accurately manipulate local drugs for on-demand tumor therapy. Biomaterials 262, 120357. 10.1016/j.biomaterials.2020.120357 32911253

[B102] Zhang R. Wang L. Wang X. Jia Q. Chen Z. Yang Z. (2020a). Acid‐induced *in vivo* assembly of gold nanoparticles for enhanced photoacoustic imaging‐guided photothermal therapy of tumors. Adv. Healthc. Mat. 9, 2000394. 10.1002/adhm.202000394 32543023

[B103] Zhang X. Tan B. Wu Y. Zhang M. Liao J. (2021). A review on hydrogels with photothermal effect in wound healing and bone tissue engineering. Polymers 13, 2100. 10.3390/polym13132100 34202237 PMC8271463

[B104] Zhang Y. Liu J. Yu Y. Chen S. Huang F. Yang C. (2020b). Enhanced radiotherapy using photothermal therapy based on dual-sensitizer of gold nanoparticles with acid-induced aggregation. Nanomedicine Nanotechnol. Biol. Med. 29, 102241. 10.1016/j.nano.2020.102241 32565227

[B105] Zhang Y. Tian S. Huang L. Li Y. Lu Y. Li H. (2022). Reactive oxygen species-responsive and Raman-traceable hydrogel combining photodynamic and immune therapy for postsurgical cancer treatment. Nat. Commun. 13, 4553–4615. 10.1038/s41467-022-32160-z 35931666 PMC9356008

[B106] Zhang Z. Lucia L. (2021). Toward synergistic reinforced graphene nanoplatelets composite hydrogels with self-healing and multi-stimuli responses. Polymer 234, 124228. 10.1016/j.polymer.2021.124228

[B107] Zhao J. Xu W. Zhao Z. Ling G. Zhang P. (2022). Intelligent nanocomposite hydrogels with simultaneous photothermal antitumor and antibacterial efficacy for cutaneous melanoma treatment. Compos. Part B Eng. 243, 110130. 10.1016/j.compositesb.2022.110130

[B108] Zheng A. Wu D. Fan M. Wang H. Liao Y. Wang Q. (2020). Injectable zwitterionic thermosensitive hydrogels with low-protein adsorption and combined effect of photothermal-chemotherapy. J. Mat. Chem. B 8, 10637–10649. 10.1039/d0tb01763a 33147312

[B109] Zhou D. Li S. Pei M. Yang H. Gu S. Tao Y. (2020a). Dopamine-modified hyaluronic acid hydrogel adhesives with fast-forming and high tissue adhesion. ACS Appl. Mat. Interfaces 12, 18225–18234. 10.1021/acsami.9b22120 32227982

[B110] Zhou L. Chen F. Hou Z. Chen Y. Luo X. (2021). Injectable self-healing CuS nanoparticle complex hydrogels with antibacterial, anti-cancer, and wound healing properties. Chem. Eng. J. 409, 128224. 10.1016/j.cej.2020.128224

[B111] Zhou L. Zhao J. Chen Y. Zheng Y. Li J. Zhao J. (2020b). MoS_2_-ALG-Fe/GOx hydrogel with Fenton catalytic activity for combined cancer photothermal, starvation, and chemodynamic therapy. Colloids Surfaces B Biointerfaces 195, 111243. 10.1016/j.colsurfb.2020.111243 32663712

